# Acute Care and Rehabilitation After Low-Trauma Hip Fractures in Older Adults—Pathways and Functional Outcomes: A Retrospective Cohort Study

**DOI:** 10.3390/jcm15208074

**Published:** 2026-10-17

**Authors:** Eva Cioffi, Karl Müller, Silviya Ivanova, Albert Antoni Fava Altes, Manuel Pfister, Valerie Frischknecht, Noora Emilia Angelva, Thimo Marcin, Matthias Wilhelm, Adrian Martinez-De la Torre, Tobias Nef, Johannes Dominik Bastian, Anke Scheel-Sailer

**Affiliations:** 1 Center for Rehabilitation & Sports Medicine, Inselspital and Berner Reha Zentrum, Bern University Hospital, University of Bern, 3010 Bern, Switzerland; k.mueller@rehabern.ch (K.M.); albertfava.cv@gmail.com (A.A.F.A.); valerie.frischknecht@insel.ch (V.F.); thimo.marcin@insel.ch (T.M.); matthias.wilhelm@insel.ch (M.W.); a.scheel-sailer@rehabern.ch (A.S.-S.); 2 Graduate School for Health Sciences, University of Bern, 3012 Bern, Switzerland; 3 Department of Orthopedic Surgery and Traumatology, Inselspital, Bern University Hospital, University of Bern, 3010 Bern, Switzerland; silviya.ivanova@insel.ch (S.I.); johannes.bastian@insel.ch (J.D.B.); 4 Faculty of Health Sciences and Medicine, University of Lucerne, 6002 Lucerne, Switzerland; manuel.pfister@paraplegie.ch (M.P.); adrian.martinez@unilu.ch (A.M.-D.l.T.); 5 Swiss Paraplegic Research, 6207 Nottwil, Switzerland; 6 Gerontechnology & Rehabilitation Group, ARTORG Center for Biomedical Engineering Research, University of Bern, 3010 Bern, Switzerland; noora.angelva@unibe.ch (N.E.A.); tobias.nef@unibe.ch (T.N.); 7 Department of Neurology, Inselspital, Bern University Hospital, University of Bern, 3010 Bern, Switzerland; 8 Centre for Rehabilitation in Global Health Systems, University of Lucerne, 6002 Lucerne, Switzerland

**Keywords:** fragility fracture, rehabilitation, orthogeriatric, pathway, functional outcome, trajectory, functional recovery, care need

## Abstract

**Background/Objectives**: This study describes, first, healthcare pathways, including inpatient rehabilitation, and, second, functional outcomes for these low-trauma hip fracture (LTHF) types (femoral neck (FN-F), trochanteric (Tr-F), and acetabulum/periprosthetic (Ac/Pp-F) fractures). **Methods**: This single-center, retrospective cohort study, 2020–2023, included patients aged ≥65 years, with FN-F, Tr-F, or Ac/Pp-F, admitted to inpatient rehabilitation. Pathways comprised acute care, inpatient rehabilitation, and temporary care homes, bounded by pre-fracture situation and discharge destination. Pairwise Standardized Mean Differences (SMDs) were calculated to determine between-group differences. **Results**: Of 294 individuals (mean age 81.0 ± 7.4 years; 191 (65%) women; 152 Tr-F, 118 FN-F, and 24 Ac/Pp-F), four main pathways were identified: (1) acute care to inpatient rehabilitation in 208 (70.7%) individuals, (2) temporary care home stays before rehabilitation in 25 (8.5%), (3) temporary care home stays after rehabilitation in 17 (5.8%), and (4) re-hospitalization in 44 (15.0%). Median pathway length was 33.0 days (IQR 26.0–40.0) for FN-F, 35.0 (IQR 29.0–46.0) for Tr-F, and 60.5 (IQR 37.0–86.5) for Ac/Pp-F. Between-group differences in pathway type were moderate to large (SMDs: FN-F vs. Tr-F = 0.47; FN-F vs. Ac/Pp-F = 1.25; Tr-F vs. Ac/Pp-F = 0.84), and for length, small to large (SMDs: FN-F vs. Tr-F = 0.36; FN-F vs. Ac/Pp-F = 1.2; Tr-F vs. Ac/Pp-F = 0.69). Overall, 246 (83.7%) returned to a permanent private living setting. A total of 88 (29.9%) required new or increased home support or care. At discharge from rehabilitation, among participants who completed the program, the *Functional Independence Measure* and the *Timed Up and Go* test showed small-to-moderate between-group differences. **Conclusions:** Individuals with FN-F, Tr-F, and Ac/Pp-F fractures follow healthcare pathways in different frequencies and have different pathway durations. The findings may support the identification of future opportunities to improve continuity of healthcare, including orthogeriatric management and specialized rehabilitation.

## 1. Introduction

Low-trauma hip fractures (LTHFs) remain a major challenge for older adults and are associated with high rates of functional decline, loss of independence, and increased mortality [1,2,3]. These fractures are anatomically heterogeneous, and are distinguished as intracapsular (femoral neck fractures (FN-Fs)) and extracapsular (trochanteric) fractures (Tr-Fs). These two are often referred to as the traditional LTHFs, while acetabular and periprosthetic fractures (Ac/Pp-Fs) represent more complex non-traditional fragility fractures. These fracture types differ substantially in surgical technique, postoperative weight-bearing restrictions, complication profiles, and rehabilitation demands [4,5,6,7,8].

Multidisciplinary orthogeriatric co-management, including rehabilitation, is recommended for older adults with fragility fractures, and has been shown to improve outcomes, including lower mortality, fewer complications, and better functional recovery [5,6,9,10]. Still, many patients do not regain their pre-fracture functioning level [1,3,11,12,13,14]. Additionally, older adults are frequently present with multiple comorbidities that must be addressed in an integrated way throughout clinical management and rehabilitation [5,15,16,17]. Recovery trajectories appear to differ between fracture types, with some evidence suggesting that patients with Tr-F tend to have worse clinical outcomes, including prolonged hospitalization and rehabilitation, compared with FN-F [18,19,20]. Outcomes following Ac/Pp-F remain less well characterized and often require more complex individualized surgical decision-making and rehabilitative management than traditional hip fractures [21,22].

To our knowledge, it is unknown whether different LTHF types follow distinct healthcare pathways, including specialized inpatient rehabilitation, and what functional outcomes are observed across LTHF types at the end of the pathway, as represented in this analysis by completion and discharge from inpatient rehabilitation.

Therefore, the primary aim of this study was to describe healthcare pathways, including inpatient rehabilitation, with respect to pathway type and length, across different LTHF types (i.e., FN-F, Tr-F, and Ac/Pp-F) in older adults. The secondary aim was to describe functional outcomes at discharge from inpatient rehabilitation among those who completed the program, across the same LTHF types. For both aims, between-group differences across the three LTHF types were investigated to assess their magnitude. In addition to FN-F and Tr-F, this study included participants with Ac/Pp-F. This heterogeneous group is underrepresented in research, and, therefore, this analysis was exploratory.

## 2. Materials and Methods

### 2.1. Study Design

This single-center, retrospective cohort study used prospectively collected routine clinical data from patients admitted to the Bern Reha Center at Heiligenschwendi Bern, Switzerland. This study was approved by the Cantonal Ethics Committee Bern (Swiss Ethics Project-ID 2024-00480, Bern Rehab Registry). This observational study follows the STROBE (Strengthening the Reporting of Observational Studies in Epidemiology) guideline [23].

### 2.2. Setting

This rehabilitation center offers inpatient rehabilitation for patients with musculoskeletal disorders, geriatric, cardiovascular, pulmonary, and internal oncological patients during the post-acute and chronic phase. The center adheres to the national quality criteria [24] and is generally accessible in the health system. Patients are referred from acute care hospitals within the greater region, ranging from regional to university hospitals.

### 2.3. Participants

Potentially eligible patients were identified from the institutional clinical database using International Classification of Diseases, 10th Revision, German Modification (ICD-10-GM) codes provided by the institutional medical coders. All patients aged ≥65 years who were admitted after 1 January 2020 and discharged before 15 September 2023 to the musculoskeletal (MSK) or MSK–geriatric inpatient rehabilitation program at this center with a primary diagnosis of acetabular fracture (S32.4) or hip fracture (S72.0–S72.9) were screened for eligibility. Participants were included if they had experienced an LTHF at the femoral neck (S72.0), trochanter (S72.1–S72.2), acetabulum (S32.4), or a periprosthetic fracture at any of these locations. Participants were excluded if they were still hospitalized at the end of the observational period (15 September 2023); if they denied retrospective use of data; if the fracture was solely at the femoral shaft (ICD codes S72.4); if they had experienced a traumatic, high-energy, or pathological fracture in an oncological condition; or if the cause of the fracture was entirely unknown. Participants were excluded if they had left the rehabilitation program against medical advice and before performing the functional outcome measures.

### 2.4. Groups

The analysis was performed for the overall cohort and stratified by fracture type, resulting in three groups: (1) *FN-F*, (2) *Tr-F*, and (3) *Ac/Pp-F*. Ac/Pp-Fs were combined into a single group, as both are considered non-traditional hip fractures that often require more specialized clinical management than the FN-F and Tr-F types. Although acetabular and periprosthetic fractures are clinically heterogeneous conditions, they were combined to achieve a sufficient sample size rather than being excluded, as both are underrepresented in the literature. This allowed these fractures to be studied in an exploratory analysis.

### 2.5. Primary Outcome

Our primary aim was the identification of (a) “*pathway type*”, (b) “*length of pathway*”, and (c) “*situation at the end of the pathway*”.

(a) Identifying the “*pathway type*” through the continuum of care, including different living and healthcare settings: Settings, from pre-fracture to outpatient setting, were mapped chronologically to reveal an individual path for each participant. Treatment before rehabilitation was documented based on reports in the clinical information system. Settings were defined according to the usual structure of living arrangements and healthcare organization in Switzerland: “*independent living*”, “*independent living with support or care*”, “*acute care hospital*”, “*inpatient rehabilitation*”, “*temporary care home*”, “*long-term care home*”, “*other*”, “*deceased*”, or “*lost to follow-up*”.

“*Independent living with support or care*” was defined as participants receiving help from a non-healthcare professional (e.g., a relative) or from a healthcare professional for self-care. “*Temporary care home*” and “*long-term care home*” are also known as old peoples’ or nursing homes. In Switzerland, individuals are usually referred to a care home if they are dependent on activities of daily living and require intensive care. Individuals with an LTHF are referred to a temporary stay in a care home if they cannot comply with weight-bearing restrictions, leading to wheelchair mobility. Physiotherapists usually assess the capacity to walk with weight-bearing restrictions during the initial acute care after surgery. If using a wheelchair is not possible in the usual living situation, a temporary stay in a care home becomes necessary. No shortage of inpatient rehabilitation beds was reported. The “*other*” category included any setting not covered by the predefined healthcare settings and distinct from the pre-fracture setting. Lastly, the “*lost to follow-up*” category was assigned if participants had unknown stays within their pathway or if their final discharge destination was not a permanent living arrangement (i.e., categories “*acute care hospital*”, “*inpatient rehabilitation*”, “*temporary care home*”, and “*other*”).

Clinical considerations, experience, and established routine primarily guided the decision for the four pathways. A stay in a temporary care home is typically approximately 3–6 weeks, depending on compliance with weight-bearing restrictions, and is often characterized by reduced mobility, which is particularly problematic for older adults. Whether and when such a stay occurs relative to inpatient rehabilitation was therefore considered a clinically meaningful distinction between the pathways P1-P3. For P4, re-hospitalization was given precedence over other pathway characteristics, because of its greater clinical relevance. Re-admissions to acute care mark an urgent medical problem or emergency, which patients and healthcare professionals want to avoid whenever possible. Therefore, the pathway categorization reflects clinically important timing of a temporary care home stay relative to inpatient rehabilitation, and the occurrence of re-hospitalization.

**Figure 1:**
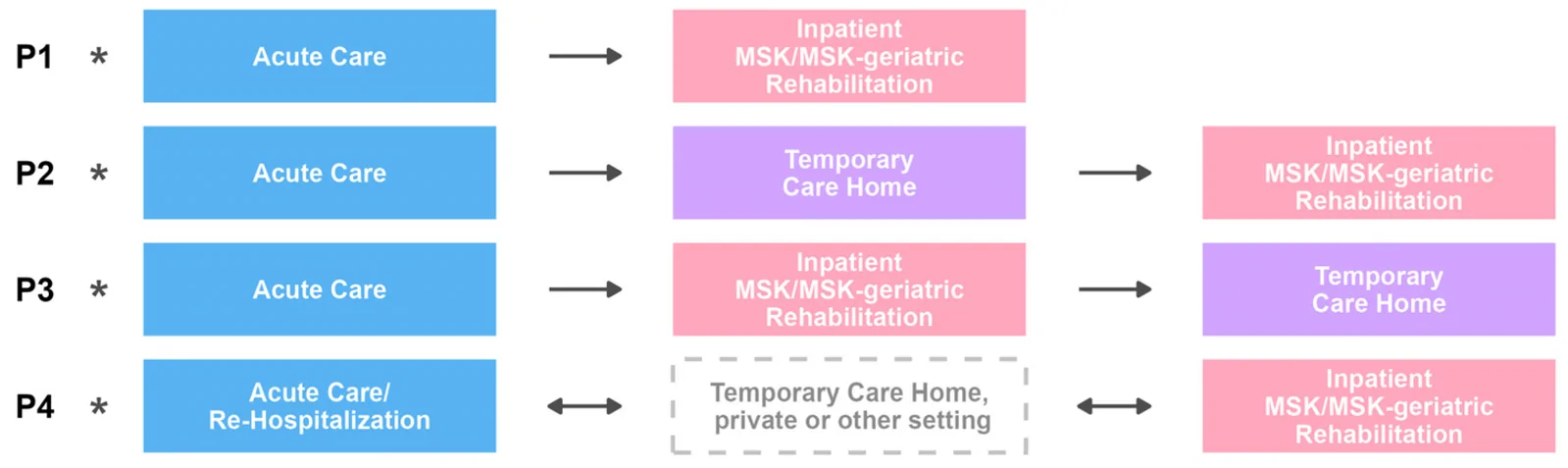
Principles of the four main healthcare pathways, starting in the acute care setting after the LTHF (indicated with an asterisk).

Individual paths consisting of the described settings above were mapped out. In a second step, four main pathways were derived from the established clinical routine, and individual paths were assigned accordingly. These four main pathways (Figure 1) were defined as follows: (1) from acute care directly to inpatient rehabilitation and discharge to outpatient setting (P1); (2) from acute care to a temporary care home, followed by inpatient rehabilitation and discharge to outpatient setting (P2); (3) from acute care to inpatient rehabilitation and then to a temporary care home (P3); and (4) all cases with hospital re-admission, irrespective of a temporary stay in a care home (P4).

(b) The total “*length of the pathway*” was calculated in days from the difference between the date of fracture (i.e., date of low-trauma fracture) and the date of discharge from inpatient rehabilitation. If the final discharge destination from rehabilitation was not a permanent living arrangement, if available, the last known date was used; otherwise, the rehabilitation discharge date was used. These participants were retained in the analysis, as their exclusion would have skewed the results.

Only for the visualizations (i.e., the bar plots), the pre-fracture living setting and the final destination were set to be the same for all participants every 3 days. Stays within the continuum of care that could not be determined were assigned with a placeholder value of 3 days and flagged as “*lost to follow-up*” in the data. This was necessary to visualize the stays. This did not affect the calculation of the pathway length, which was determined as the difference between the dates, as described above.

(c) The “*situation at the end of the pathway*” was derived from the settings described above and consisted of four categories: “*unchanged care or support level*”; “*new or increased home-based support or care*”; “*new institutional care*” compared to the pre-fracture level; and the fourth category, “*other*”, which included deceased or lost to follow-up individuals.

All four pathway outcome categories were derived from the settings defined above (as described in (*a*) above). The first two categories were assigned to participants discharged to a permanent living arrangement, based on whether their support and care needs had changed compared to the pre-fracture setting. The “*unchanged care or support level*” category included participants who had previously lived in a permanent living setting with or without support or care, and returned to the same situation with no change in support or care needs. The “*new or increased home-based support or care*” category included participants who had initially lived independently but were discharged to a setting requiring greater support or care (i.e., transitioned from “*independent living*” to “*independent living with support or care*”). “*New institutional care*” referred to permanent living in a long-term care home, such as a residential or nursing home. Participants who had died or whose final destination was not a permanent living setting were assigned to the “*other*” (which included the categories “*deceased*” or “*lost to follow-up*”) category.

### 2.6. Secondary Outcomes

Our secondary outcomes were functioning at discharge from inpatient rehabilitation. They included self-care and mobility: (d) the degree of independence, measured with the “*Functional Independence Measure*” (FIM), and (e) mobility, measured with the “*Timed Up and Go*” (TUG) test [25,26].

For (d) and (e), FIM and TUG were both assessed at discharge from inpatient rehabilitation and analyzed for participants who completed the rehabilitation program and were discharged to a permanent living arrangement. Both measures were extracted automatically (using custom Structured Query Language scripts) from the medical information system. Nurses assessed the FIM, whereas TUG was assessed by physiotherapists, as defined by the internal standard-of-care procedures. Additionally, supportive gait devices were reported, which have been used during the TUG test.

### 2.7. Measurements and Variables

Baseline characteristics and variables (see Table 1 in the Result Section) refer to the pre-fracture situation and include sociodemographic variables such as “*age*”, “*sex*”, and “*pre-fracture living setting*”. Age was calculated at the time of the LTHF. Sex was recorded as “*male*” or “*female*”. Age and sex were extracted automatically. The “*pre-fracture living setting*” variable used the same setting categories as those described above in (a) for the pathway. Further, the pre-fracture “*Clinical Frailty Scale*” (CFS) was retrospectively assessed using documentation from healthcare professionals in the medical information system [27,28]. In our cohort, only levels 1 (“*very fit*”) to 7 (“*severely frail*”) of the CFS were represented, while no participant was assessed at level 8 (“*very severe frail*”) or level 9 (“*terminally ill*”). Pre-fracture gait and balance disturbances and previous low-trauma fractures were collected by reviewing the medical diagnosis list, each recorded as yes, no, or unknown, covering, “*presence of previous falls*”, “*experience of gait and balance disturbances*”, and “*experienced previous low-trauma fracture(s) at the hip, pelvis, proximal femur, or femoral shaft*”. Severity of illness was assessed using the “*Cumulative Illness Rating Scale*” (CIRS), completed by physicians at the time of admission to inpatient rehabilitation [29]. The CIRS values were automatically extracted from the medical information system. “*Body Mass Index*” (BMI) was calculated using weight and height measured by healthcare professionals at admission to rehabilitation, ensuring reliable values.

Information regarding “*acute care treatment schema*” and “*prescribed weight-bearing*” was extracted from medical records. Prescribed weight-bearing was collapsed into three categories: “*no weight-bearing*” (including bed-to-wheelchair only and toe-touch, i.e., 5–10 kg), “*partial weight-bearing*” (15–20 kg, 30 kg, half body weight, and partial weight-bearing of unspecified amount), and “*full weight-bearing or weight-bearing as tolerated (WBAT)*”. Rehabilitation discipline was categorized as “*standard musculoskeletal*” (MSK) or “*specialized MSK–geriatric*”, with assignment based on national requirements [30]. Additional patient and healthcare descriptives, as well as an extended methods section, are available in the Appendix A and Appendix B. Data were collected and managed using REDCap^©^ electronic data-capture tools hosted at the University of Bern [31,32].

### 2.8. Data Analysis

Variables were analyzed by calculating frequencies (for categorical variables) or summary statistics (for continuous variables), both overall and for each of the three fracture groups. For normally distributed continuous data, we calculated the mean and standard deviation (SD). For non-normal data, the median and the interquartile range (IQR) are at the 25th (Q1) and 75th (Q3) percentiles. Missing values are reported only for variables with incomplete data and are reported as counts.

To assess the magnitude of difference between groups on descriptive variables, the pairwise *Standardized Mean Difference* (SMD) was used. The aim was to assess the magnitude of differences rather than rely on significance thresholds, as with *p*-values, thereby avoiding problems of multiple hypothesis testing. Unlike significance tests, standardized differences are not directly influenced by the sample size, as the formulas below show [33,34], although they may be estimated less precisely in smaller samples. This makes them suitable for comparing groups with imbalanced sample sizes, as in this study. Because the Ac/Pp-F group was a heterogenous group and much smaller than the FN-F and TR-F groups, analyses involving this group were exploratory. Standardized differences are calculated using different formulas depending on the data type (i.e., continuous, binary, or multinomial categorical variables). We used *R* version 4.5.3 (11 March 2026). SMD values were calculated using the R package *smd* (version 0.8.0, 12 February 2025) [35], which implements the SMD’s calculations based on Yang and Dalton (2012) [33] for different data types as follows:

1. *Continuous variables*

SMDs, *d*, for continuous variables were calculated based on the formula

$$d=\frac{{\bar{X}}_{1}-{\bar{X}}_{2}}{\sqrt{\frac{s_{1}^{2}+s_{2}^{2}}{2}}}$$

where ${\bar{X}}_{1}$ and ${\bar{X}}_{2}$ are the variable’s sample mean for each group, and $s_{1}^{2}$ and $s_{2}^{2}$ are the sample variances.

2. *Binary categorical variables*

Standardized differences, *d*, for binary variables were calculated based on the formula

$$d=\frac{\left({\hat{P}}_{1}-{\hat{P}}_{2}\right)}{\sqrt{\frac{\left({\hat{P}}_{1}(1-{\hat{P}}_{1})+{\hat{P}}_{2}(1-{\hat{P}}_{2}\right)}{2}}}$$

where ${\hat{P}}_{1}$ and ${\hat{P}}_{2}$ are the variables’ proportion for each group.

3. *Multinomial categorical variables*

Standardized difference, *d*, for categorical variables with >2 levels (i.e., with *K* levels) was calculated based on the proposed approach by Dalton (2008) [34] by using a multivariate Mahalanobis distance method:

$$d=\sqrt{{\left(G_{1}-G_{2}\right)}^{\prime }S^{-1}(G_{1}-G_{2})}$$

where *G*_1_ and *G*_2_ are the vectors of the proportions for each group, and *S* is a covariance matrix. Further details on the covariance matrix and calculations can be found in the original research article by Young and Dalton (2012) [33].

In addition to the SMD values, *Confidence Intervals* (CIs) are reported based on Hedges and Olkin (1985) [36]. The 95% CI for standardized difference, *d*, is defined as

d±1.96×σ[d]

with

$$\sigma \left[d\right]=\sqrt{\frac{n_{1}+n_{2}}{n_{1}\times n_{2}}+\frac{d^{2}}{2(n_{1}+n_{2})}}$$

where *n*_1_ and *n*_2_ are the sample sizes for each group.

Effect sizes were interpreted using the proposed thresholds for gerontological research by Brydges (2019), as Cohen’s thresholds have been shown to overestimate effect sizes in such research [37]. Because this study was conducted with older adults, Brydges’s (2019) threshold was considered more appropriate. SMD values ≤0.15, 0.40, and >0.75 represented *small*, *medium*, and *large* effects in standardized differences, respectively. An SMD value below 0.15 was interpreted as *negligible*. Tables were computed using the R *gtsummary* package version 2.5.0. [38].

## 3. Results

### 3.1. Participants and Baseline Characteristics

**Figure 2:**
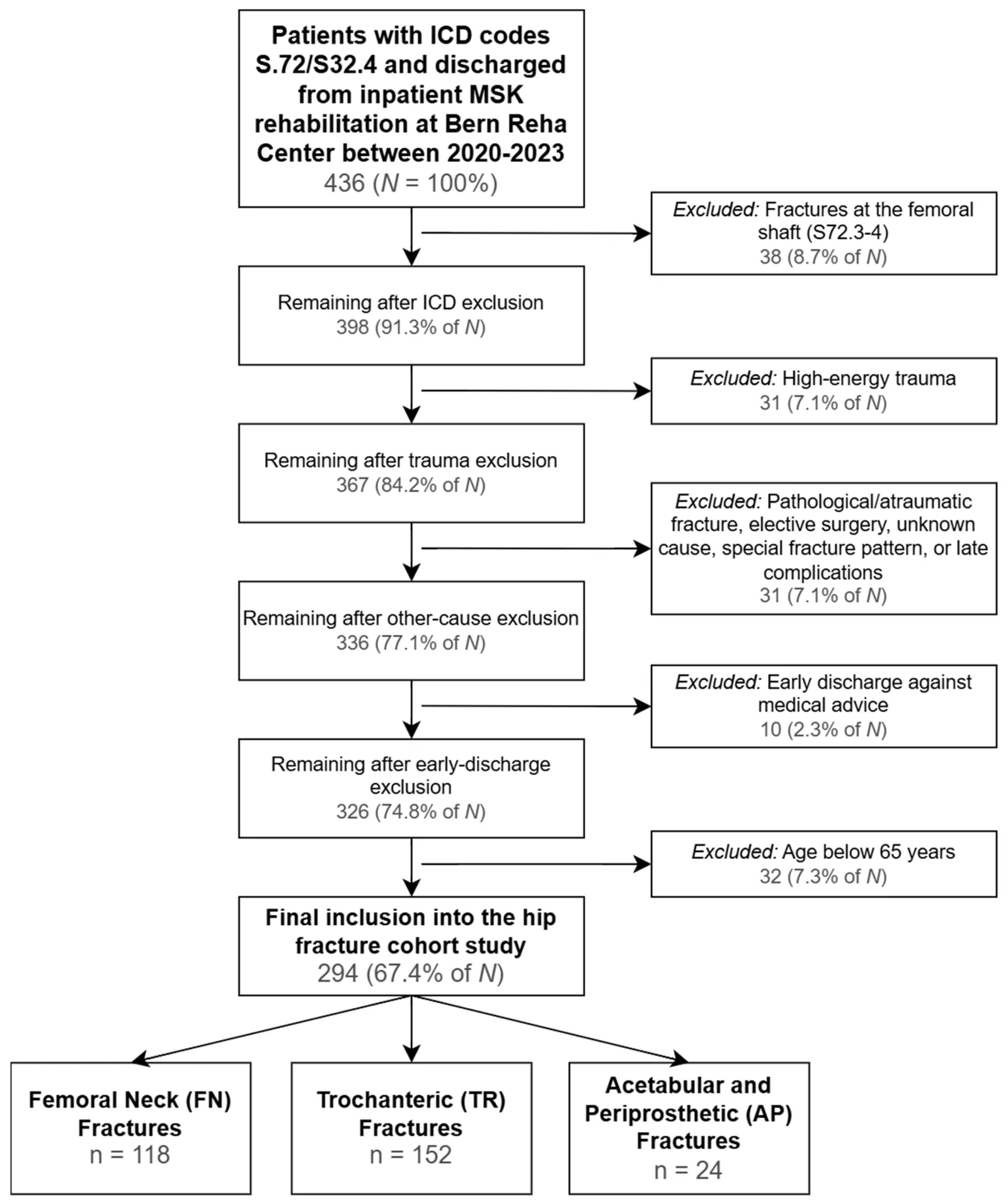
The flowchart shows participant selection for the cohort study. Patients admitted to inpatient musculoskeletal (MSK) rehabilitation following a hip fracture (ICD codes S72/S32.4) were screened for eligibility. Exclusion criteria were applied in sequence, and the final hip fracture cohort comprised 294 participants. Three groups were defined by hip fragility fracture types: femoral neck (FN-F), trochanteric (Tr-F), and acetabular/periprosthetic (Ac/Pp-F) fractures. *N* denotes the initial number of patients who were screened, and *n* denotes the number of participants in the included groups.

Initially, 436 patients discharged from inpatient rehabilitation between 2020 and 2023 were screened for eligibility. After excluding 142 individuals, 294 participants were included in the final analysis (Figure 2).

The selected study cohort included participants who were admitted to inpatient rehabilitation at some point in their healthcare pathway: 118 in the FN-F group, 152 in the Tr-F group, and 24 in the Ac/Pp-F group, which consisted of six individuals with an acetabular fracture and 18 with a periprosthetic fracture. Patients with an acetabular fracture had a mean age of 76.7 ± 8.6 years; two (33.3%) were female, four (66.7%) lived in an independent living setting before the fracture, and none lived in a long-term care home. Patients with a periprosthetic fracture were older on average (80.4 ± 7.7 years), more female individuals (12 (66.7%)), 12 (66.7%) lived in an independent living setting, and one (5.6%) lived in a long-term care home. Separated key descriptives for participants with the acetabular and periprosthetic fractures are provided in the Appendix A Table A8. Except for the number of surgeries in the Ac/Pp-F group, we observed no relevant SMD differences for type and length of pathway, and the functional outcomes (Appendix A Table A9).

For the baseline descriptives, between-group differences were mostly small across the three LTHF groups (Table 1 and Appendix A Table A1, Table A2 and Table A3), as indicated by the SMD values. A medium effect was observed for the CFS, where categories 5–7 reflect the need for support or care: lowest for the FN-F group with 53 participants (44.9%), followed by the Ac/Pp-F group (12, 50.0%), and the Tr-F group with 84 (55.0%) participants (Table 1). The wide 95% CIs indicate considerable uncertainty for some SMDs, particularly for comparisons involving the Ac/Pp-F group. This also applies to the other tables, including the tables in the appendix (Appendix A Table A1, Table A2, Table A3, Table A4, Table A5, Table A6 and Table A7).

**Table 1:** Baseline characteristics of the study cohort, overall, and stratified for each of the three hip fragility fracture groups. All variables refer to the pre-fracture time, except the Cumulative Illness Rating Scale (CIRS) and Body Mass Index, which were assessed at admission to inpatient rehabilitation.

		Overall	FN-F	Tr-F	Ac/Pp-F	SMD (95% CI)^2^		
		N = 294 (%) ^1^	N = 118 (%) ^1^	N = 152 (%) ^1^	N = 24 (%) ^1^	FN-F vs. Tr-F ^2^	FN-F vs. Ac/Pp-F ^2^	Tr-F vs. Ac/Pp-F ^2^
**Age**		81.0 ± 7.4	80.8 ± 7.0	81.5 ± 7.6	79.5 ± 7.9	0.10 (−0.14, 0.34)	0.18 (−0.26, 0.62)	0.26 (−0.17, 0.70)
**Sex**	Women	191 (65.0)	79 (66.9)	98 (64.5)	14 (58.3)	0.05 (−0.19, 0.29)	0.18 (−0.26, 0.62)	0.13 (−0.30, 0.56)
	Men	103 (35.0)	39 (33.1)	54 (35.5)	10 (41.7)			
**Pre-fracture living setting**	Independent living	238 (81.0)	99 (83.9)	123 (80.9)	16 (66.7)	0.20 (−0.05, 0.44)	0.41 (−0.03, 0.85)	0.36 (−0.07, 0.79)
	Independent living with support or care	51 (17.3)	16 (13.6)	28 (18.4)	7 (29.2)			
	Long-term care home	5 (1.7)	3 (2.5)	1 (0.7)	1 (4.2)			
**Clinical Frailty Scale** ^3^	1—Very fit	4 (1.4)	3 (2.5)	0 (0.0)	1 (4.2)	0.38 (0.14, 0.62)	0.54 (0.10, 0.98)	0.68 (0.24, 1.12)
	2—Well	46 (15.6)	22 (18.6)	21 (13.8)	3 (12.5)			
	3—Managing well	27 (9.2)	9 (7.6)	15 (9.9)	3 (12.5)			
	4—Vulnerable	68 (23.1)	31 (26.3)	32 (21.1)	5 (20.8)			
	5—Mildly frail	105 (35.7)	40 (33.9)	59 (38.8)	6 (25.0)			
	6—Moderately frail	33 (11.2)	9 (7.6)	22 (14.5)	2 (8.3)			
	7—Severely frail	11 (3.7)	4 (3.4)	3 (2.0)	4 (16.7)			
**Presence of previous falls**		151 (51.4)	62 (52.5)	78 (51.3)	11 (45.8)	0.02 (−0.22, 0.27)	0.13 (−0.30, 0.57)	0.11 (−0.32, 0.54)
**Experience of gait and balance disturbances**		53 (18.0)	23 (19.5)	26 (17.1)	4 (16.7)	0.06 (−0.18, 0.30)	0.07 (−0.37, 0.51)	0.01 (−0.42, 0.44)
**Experienced previous low-trauma fracture(s) at the hip,** **pelvis, proximal femur, or femoral shaft** *—Unknown*		45 (15.5) *4*	15 (12.8) *5*	20 (13.3) *2*	10 (43.5) *1*	0.02 (−0.23, 0.26)	0.73 (0.27, 1.18)	0.71 (0.26, 1.15)
**Cumulative Illness Rating Scale—Total Score**		20.8 ± 5.5	20.3 ± 5.6	21.2 ± 5.6	20.8 ± 4.4	0.16 (−0.08, 0.40)	0.10 (−0.34, 0.54)	0.08 (−0.35, 0.51)
**Body Mass Index**		25.3 ± 5.1	24.7 ± 4.4	25.6 ± 5.6	26.0 ± 5.0	0.19 (−0.05, 0.43)	0.29 (−0.15, 0.73)	0.08 (−0.36, 0.51)

^1^ Mean ± SD; *n* (%). ^2^ Pairwise Standardized Mean Difference (SMD) with 95% Confidence Interval (CI) ^3^ For the Clinical Frailty Scale (CFS), the last two levels (*8—very severely frail;* and *9—terminally ill*) were left out because there was no individual assigned to these two categories.

### 3.2. Type of Pathway

All individual paths were mapped out, and 40 unique sequencies were identified (Appendix A Table A10). For the four main pathways, the most direct pathway, P1, was most common in the FN-F group (80.5%), less common in the Tr-F group (67.8%), and least common in the Ac/Pp-F group (41.7%), with moderate-to-large between-group effects, as indicated with the SMDs (Table 2). Conversely, temporary care-home stays (P2 and P3) followed the opposite pattern. P4 with re-hospitalization occurred similarly across all groups, but the least in the Ac/Pp-F group (Table 2). SMDs involving the Ac/Pp-F group had wide CIs, indicating considerable uncertainty in these estimates. Detailed separate frequencies of pathway types for acetabular and periprosthetic fractures are shown in Appendix A Table A8 and Table A9.

**Figure 3:**
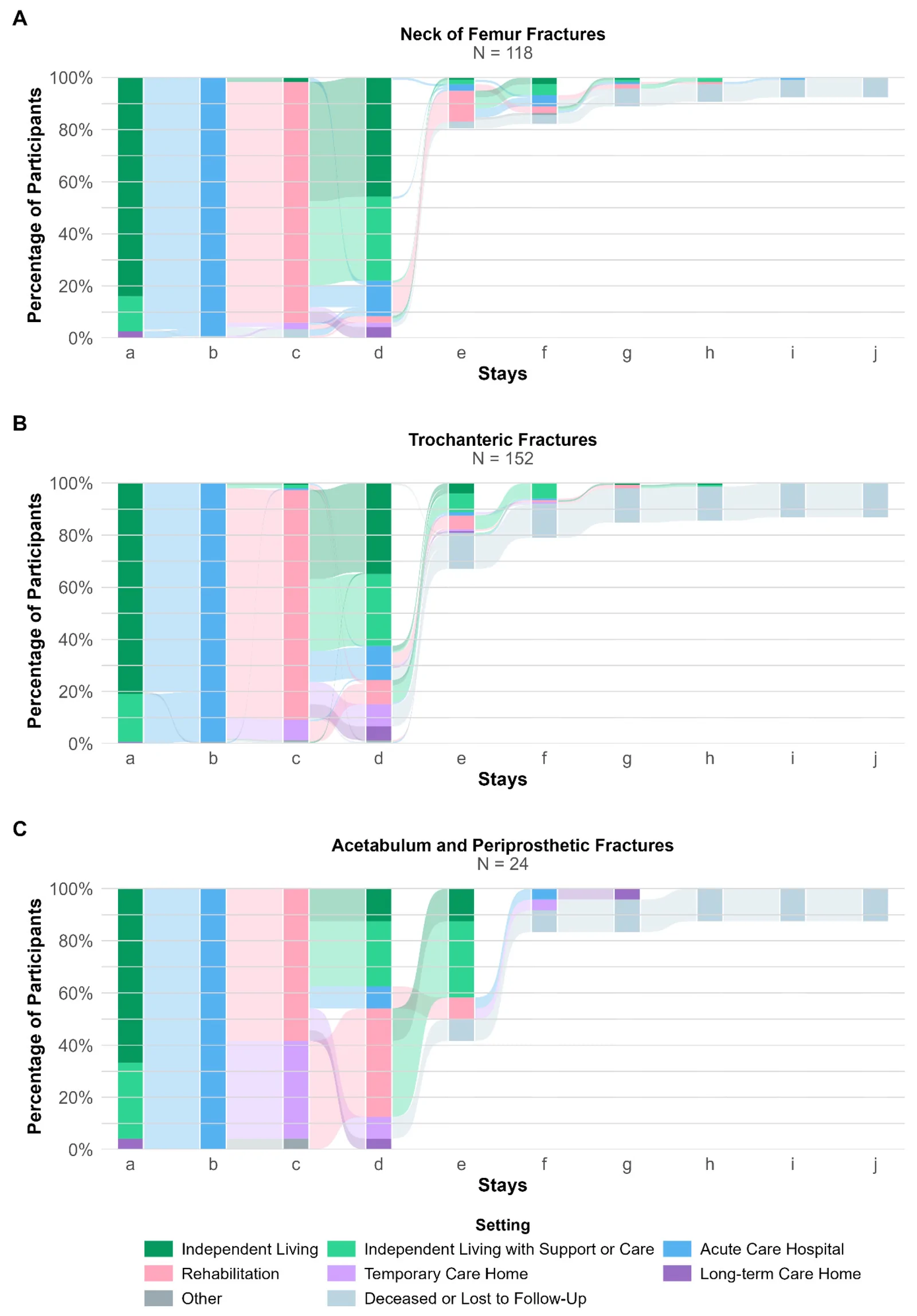
(**A**–**C**) Flow plots showing participants’ transitions from the pre-fracture situation through the different healthcare settings (from left to right), including an acute care hospital, an inpatient rehabilitation facility, and a temporary care home. The transitions are stratified for the three LTHF (Low-Trauma Hip Fracture) types. On the x-axis, (a) is the permanent pre-fracture living setting, (b) is the acute care setting for most participants, and (c–j) are the following different settings participants move through until discharged or lost to follow-up.

The flow plots (Figure 3A–C) show that, in all three groups, the pathways have formed a continuum of care, with direct transitions between healthcare settings. Most individuals in the FN-F and Tr-F groups returned to an outpatient setting with the 3rd transition, whereas for the Ac/Pp-F group, this occurred with the 3rd and 4th transitions.

### 3.3. Length of Pathway

**Figure 4:**
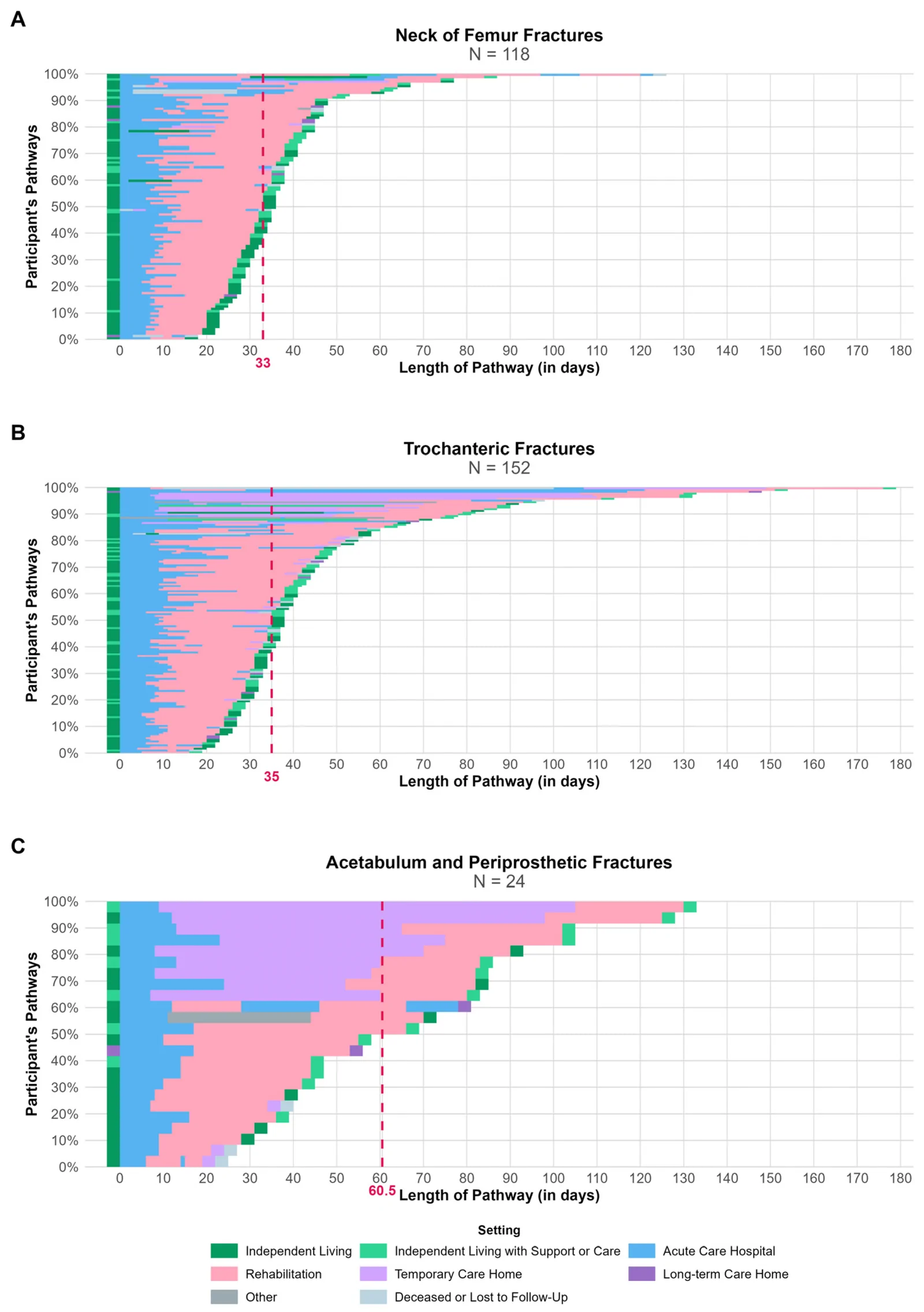
(**A**–**C**) Bar plots showing participants’ pathways with their corresponding length of stay in each setting and stratified for the three LTHF (Low-Trauma Hip Fracture) types. One horizontal line represents one participant. A dashed red line indicates the median length of the pathway for each group. On the y-axis, the pathways are ordered by participants’ total pathway length. Day 0 marks the entry into the continuum of care. The pre-fracture setting (shown before day 0), lost-to-follow-up situations, and the final setting were each assigned 3 days for visualization purposes only.

Visible from Table 2 and the bar plots (Figure 4A–C), longer pathways seemed to co-occur with temporary care-home stays and re-admissions to the acute care hospitals. Detailed frequencies of pathway length for acetabular and periprosthetic fractures are shown in Appendix A Table A8.

### 3.4. Living Situation at the End of the Pathway

Overall, 246 participants (83.7%) returned to a permanent private living setting irrespective of support or care needs (Table 2). New or increased home-based support or care, as compared to the pre-fracture situation, was detected in 88 (29.9%) individuals (Table 2).

The proportion returning to an independent living setting was highest among patients with FN-F (58, 49.2%) and lower among patients with Tr-F (59, 38.8%) and Ac/Pp-F (6, 25.0%). Independent living with support or care showed the opposite pattern (FN-F 46, 39.0%; Tr-F 64, 42.1%; Ac/Pp-F 13, 54.2%). Living in a long-term care home was proportionally more common in the Ac/Pp-F group, with two individuals (8.3%), and less common in the FN-F group, five individuals (4.2%), with nine individuals (5.9%) in the Tr-F group.

Reasons for lost-to-follow-up situations were discharge to a temporary care home (17, 5.8%), acute care hospital admission (12, 4.1%), discharge to another location (1, 0.3%), or death (2, 0.7%). For a detailed description of the pathway destination, see also Appendix A Table A6.

### 3.5. Functioning Outcomes Selfcare and Mobility

Among participants who completed the inpatient rehabilitation program, the degree of independence at discharge, as measured by the FIM, was highest in the FN-F and Tr-F group and lower in the Ac/Pp-F group. The FIM motor and FIM cognition sub-scores showed a similar pattern to the total FIM (Table 3).

Median TUG times at discharge were slightly higher across groups, indicating a slower gait speed, especially for the Ac/Pp-F group (FF-F < Tr-F < Ac/Pp-F; Table 3). Most participants required a supportive gait device during the test. The use of supportive devices differed from small to moderately between groups, with a rollator or walking frame used more frequently in the Tr-F group (36.0% and 10.5%, respectively) and the Ac/Pp-F group (43.8% rollator), compared to the FN-F group, which used one or two crutches most frequently (65%) (Table 3).

## 4. Discussion

This cohort study examined, first, the type and length of healthcare pathways in older adults undergoing inpatient rehabilitation after an LTHF, and, second, functional outcomes at discharge among those who completed inpatient rehabilitation. While no large differences were detected for baseline patient characteristics across the LTHF types, except for moderate differences in pre-fracture frailty, differences emerged in healthcare pathways with respect to type and length. Patients in the Ac/Pp-F group followed more frequently complex pathways involving multiple healthcare settings and experienced the longest overall treatment durations. Furthermore, the LTHF types differed little for the situation at the end of the pathway. This study observes that, overall, 83.7% returned to a permanent private living setting, and 29.9% required new or increased home-based support or care compared with their pre-fracture situation. Lastly, functional outcomes measured at rehabilitation discharge among completers showed relatively small observed between-group differences, with outcomes gradually poorer in the Ac/Pp-F, followed by the Tr-F group. These findings should be interpreted with caution, as the Ac/Pp-F group was small and heterogeneous. Additionally, CIs indicate uncertainty for some SMD values.

Extended pathways frequently involved temporary care-home stays. From clinical experience, this is often necessary to safely manage more restrictive weight-bearing protocols in patients who were unable to meet weight-bearing restrictions or lacked a wheelchair-accessible home environment. Other clinical factors may also influence the decision to transfer to a temporary care home. Longer waiting times until rehabilitation admission were not reported in our cohort. Temporary care-home stays occurred across all fracture groups with different frequencies. In Switzerland, such placements frequently represent a significant out-of-pocket financial burden on the patients. Furthermore, the low intensity of physiotherapy typically provided in temporary care homes (often frequently limited to one or two sessions per week) poses a substantial risk of further muscle loss and functional decline, particularly in a population who already has or is at risk of sarcopenia [39,40]. Especially for the Ac/Pp-F group, we observed slightly poorer functional outcomes, e.g., longer TUG times and greater reliance on walking aids, which could reflect reduced mobility training during the interim placement. This interpretation should be treated with caution, as participants discharged to a temporary care home may differ from other groups. Longer TUG times and greater reliance on walking aids in some groups are compatible with that interpretation, but they were not analyzed in this study as effects of temporary nursing-home stays. Therefore, further research is needed to clarify this suspected association. 

However, in the present study, healthcare pathways differed in frequency across LTHF types, as did postoperative weight-bearing protocols. An early surgical-risk assessment determines postoperative weight-bearing restrictions and may have strongly influenced the subsequent pathway. Additionally, rehabilitation admission during the observation period was possible only once patients could mobilize with their respective weight-bearing restrictions. Modern approaches, such as anti-gravity treadmill training and adapted wheelchair-based group activities, have been proposed to enable earlier rehabilitation under weight-bearing restrictions to avoid a stay in a temporary care home and subsequently improve functional outcomes. These approaches were not evaluated in the present study and remain questions for future research, as does the interplay between pathway type and length and weight-bearing restrictions.

Re-hospitalizations (pathway P4) occurred in all LTHF groups with different frequencies. Collectively, this reinforces the importance of understanding all three LTHF types within a broader risk constellation, as proposed by Bissoto et al. (2026) with the concept of an “Orthogeriatric Fracture Syndrome” [9]. However, re-hospitalization was higher among patients with FN-F and Tr-F than among those with an Ac/Pp-F. This may reflect that individuals with an Ac/Pp-F are often managed with more cautious weight-bearing strategies and longer periods of protected mobilization [7]. Periprosthetic fractures, in particular, appear to carry an elevated risk of rehospitalization [41]. Recent Scottish national audit data confirm that these injuries are associated with substantial one-year mortality and functional decline, reinforcing the need for tailored multidisciplinary pathways comparable to those established for native hip fractures [22]. Furthermore, in Switzerland, Ac/Pp-F fractures are typically managed at specialized university hospitals with established orthogeriatric care models. In contrast, FN-F and Tr-F fractures can often be treated at regional hospitals, which offer less integrated care [42]. Evidence linking orthogeriatric care to reduced re-admissions cannot be established with this study, and evidence also remains limited in research [43].

Length-of-stay patterns further illustrate differences in healthcare settings. Acute hospital stays in Switzerland are typically short (4–8 days according to diagnosis-related group recommendations) with subsequent transfers to dedicated rehabilitation centers [44]. In contrast, acute care hospital and rehabilitation stays vary internationally; for example, Japan reports 20–40 days plus rehabilitation, top UK centers achieve about 15–21 days total, and US patients typically spend 25–30 days or longer in Skilled Nursing Facilities following brief acute hospital admissions [45,46,47]. Comparison between the different healthcare systems, therefore, remains difficult due to different reporting structures and definitions of rehabilitation services or care models. However, this study observed that, overall, 83.7% returned to a private living setting, co-occurring with relatively long overall pathway durations, particularly among patients within the Ac/Pp-F group. A recent study by Cogan et al. (2020) showed that, with shorter stays in inpatient rehabilitation, additional burden of care may be transferred to the outpatient setting [48]. The Ac/Pp-F group required the most intensive care in terms of both pathway length and number of healthcare transitions. Acetabular and periprosthetic fractures in older adults pose a particular surgical challenge, necessitating individualized decisions between osteosynthesis alone or combined with acute total hip arthroplasty, depending on patient reserve, functional demand, frailty, co-morbidity burden, bone quality, and fracture characteristics to achieve the best possible postoperative outcomes, including functioning and lower complication rates [21,49,50]. Surgical management predominated in the FN-F, typically with hemi- or total hip arthroplasty, and in the Tr-F group with intramedullary nailing. Unlike for FN-F and Tr-F, treatment protocols for Ac/Pp-F are not yet well established. Especially, the Ac/Pp-F fractures are recognized as more complex and suspected to have become more common in recent years [49,50]. The Ac/Pp-F group is clinically heterogeneous and warrants further investigation.

Returning home is very important for older adults [51], and most participants in our selected cohort, which includes older adults being admitted to inpatient rehabilitation, were observed to do so. In international comparisons, for example, our observed Swiss cohort was slightly younger than individuals in a Japanese hip fracture cohort (about 82–86 years), in which about 81% remained at home, and 12% moved to care facilities within the first 6 months post-injury [52,53]. Using United Kingdom national data, mean ages of individuals with LTHF were around 82–83 years and return-to-original-residence rates of approximately 70% at 120 days [54,55]. However, returning home does not necessarily mean regaining pre-fracture functioning, and many patients may still require continued support and rehabilitation after discharge. We observed that almost 30% required new or increased home-based support or care, and most patients still had functional limitations at discharge from rehabilitation. This could point to the need for continued rehabilitation beyond inpatient rehabilitation discharge. Findings from a large European study across 45 countries show that rehabilitation services for older adults are often unevenly available across different settings. While Switzerland seems to perform strongly on service responsiveness and availability of inpatient rehabilitation, important gaps in continuity of rehabilitation in other settings, such as the community, remain [56]. Whether longer pathways contribute to a return to a private living setting could not be tested with this descriptive design and remains an important question for future studies.

### Strengths and Limitations

The most important strength is that we were able to collect comprehensive data sets in this register for four different hip fracture types based on the recommended data collection for quality measurement in the national system [57,58,59]. An important limitation is that only patients who were admitted to inpatient rehabilitation were included. Patients discharged directly to long-term care, those considered too frail for intensive rehabilitation, or those who died in the acute phase were not captured. Consequently, the observed proportion of return to a private living setting (83.7%) and the functional outcomes at rehabilitation discharge likely represent a more favorable subgroup of older adults with LTHF.

Secondly, follow-up ended at discharge from inpatient rehabilitation. This precludes conclusions about longer-term functional recovery, persistent care needs, or late complications, particularly in individuals who were discharged to a non-permanent setting (e.g., a long-term care home or acute care hospital) and were subsequently lost to follow-up.

Thirdly, the analysis of functional outcomes was limited to participants who completed the inpatient rehabilitation program. Participants who did not complete rehabilitation could not be included, potentially introducing selection bias. The proportion of non-completers was slightly higher in the Tr-F and Ac/Pp-F groups.

Fourthly, the sample size of the Ac/Pp-F group was small (24 individuals), limiting the precision of between-group comparisons and the overall generalizability of findings for this subgroup as indicated by the SMDs’ CI. Additionally, the Ac/Pp-F group is clinically heterogeneous, and opposing differences within this group may have cancelled each other out, meaning that the absence of observed differences to the two other groups (FN-F and Tr-F), therefore, may not imply equivalence. Related to this, heterogeneity increased the within-group variance for SMD values and reduced the size of the effect. We investigated SMDs including only the periprosthetic fractures, which yielded SMDs similar to those of the combined Ac/Pp-F group, except for the number of surgeries. However, this analysis does not resolve the limitation, as the restricted subgroup was even smaller, and residual heterogeneity cannot be excluded. This is a major limitation for this subgroup, and as the analysis was exploratory, the results should be interpreted with caution.

Lastly, this was a single-center cohort study. Although the setting is representative of Swiss healthcare structures and treatment organization, generalizability to other systems may be limited.

## 5. Conclusions

This observational study describes current orthogeriatric healthcare pathways involving specialized inpatient rehabilitation for different LTHF types in older adults. The findings show that LTHFs are not a uniform clinical entity: patients with FN-F, Tr-F, and Ac/Pp-F fractures follow healthcare pathways in different frequencies and have different pathway durations. The findings describe the current situation and may support the identification of future opportunities to improve continuity of healthcare and to reduce transitions between healthcare settings. Rehabilitation could also be further developed to be offered at varying intensities, for example, for individuals with a LTHF unable to comply with weight-bearing restrictions, and extended beyond discharge. Above all, further research is needed to better understand the interplay between pathway type and length, weight-bearing restrictions, returning home, care needs, and functional outcomes. Additionally, LTHF subgroups, particularly the Ac/Pp-F group and high-risk subgroups, should be characterized in more detail.

## Figures and Tables

**Table 2:** Pathway characteristics overall and stratified for each hip fragility fracture group.

		Overall	FN-F	Tr-F	Ac/Pp-F	SMD (95% CI) ^2^		
		N = 294 (%) ^1^	N = 118 (%) ^1^	N = 152 (%) ^1^	N = 24 (%) ^1^	FN-F vs. Tr-F	FN-F vs. Ac/Pp-F	Tr-F vs. Ac/Pp-F
**Pathway type**	P1—Acute care to rehabilitation	208 (70.7)	95 (80.5)	103 (67.8)	10 (41.7)	0.47 (0.22, 0.71)	1.25 (0.79, 1.71)	0.84 (0.40, 1.28)
	P2—Acute care to temp. ^3^ care home to rehabilitation	25 (8.5)	2 (1.7)	13 (8.6)	10 (41.7)			
	P3—Acute care to rehabilitation to temp. ^3^ care home	17 (5.8)	2 (1.7)	13 (8.6)	2 (8.3)			
	P4—All with re-hospitalization	44 (15.0)	19 (16.1)	23 (15.1)	2 (8.3)			
**Length of pathway** (from fracture to pathway destination in days)		35.0 [28.0, 45.0]	33.0 [26.0, 40.0]	35.0 [29.0, 46.0]	60.5 [37.0, 86.5]	0.36 (0.12, 0.60)	1.2 (0.74, 1.66)	0.69 (0.26, 1.13)
**Acute Care Treatment Schema**	Directly surgery	270 (91.8)	104 (88.1)	148 (97.4)	18 (75.0)	0.4 (0.15, 0.64)	0.77 (0.32, 1.22)	0.72 (0.29, 1.16)
	Initial conservative therapy followed by surgery	11 (3.7)	10 (8.5)	1 (0.7)	0 (0.0)			
	Only conservative therapy	13 (4.4)	4 (3.4)	3 (2.0)	6 (25.0)			
**Reason for admission of a second acute care stay** ^4,5^	Hip associated hematoma or seroma	3 (6.8)	2 (10.5)	1 (4.3)	0 (0.0)			
	Failure of osteosynthesis or endoprosthesis	3 (6.8)	0 (0.0)	3 (13.0)	0 (0.0)			
	Periprosthetic fractures	6 (13.6)	2 (10.5)	4 (17.4)	0 (0.0)			
	Other complications at the hip	9 (20.5)	7 (36.8)	2 (8.7)	0 (0.0)			
	Other reasons requiring acute care	23 (52.3)	8 (42.1)	13 (56.5)	2 (100.0)			
	*No acute care re-admission*	*250*	*99*	*129*	*22*			
**Reason for admission of a third acute care stay** ^4,5^	Periprosthetic fractures	1 (16.7)	1 (20.0)	0 (0.0)	0 (0.0)			
	Other complications at the hip	3 (50.0)	3 (60.0)	0 (0.0)	0 (0.0)			
	Other reasons requiring acute care	2 (33.3)	1 (20.0)	0 (0.0)	1 (100.0)			
	*No 2nd acute care re-admission*	*288*	*113*	*152*	*23*			
**Prescribed weight-bearing**	No weight-bearing	20 (6.8)	2 (1.7)	11 (7.2)	7 (29.2)	0.4 (0.16, 0.65)	2.67 (2.13, 3.21)	1.75 (1.29, 2.22)
	Partial weight-bearing	73 (24.8)	18 (15.3)	39 (25.7)	16 (66.7)			
	Full weight-bearing or WBAT ^3^	201 (68.4)	98 (83.1)	102 (67.1)	1 (4.2)			
**Rehabilitation discipline**	Standard MSK ^3^	171 (58.2)	69 (58.5)	88 (57.9)	14 (58.3)	0.01 (−0.23, 0.25)	0.0 (−0.44, 0.44)	0.01 (−0.42, 0.44)
	Specialized MSK ^3^–geriatric	123 (41.8)	49 (41.5)	64 (42.1)	10 (41.7)			
**Destination is a private living setting**		246 (83.7)	104 (88.1)	123 (80.9)	19 (79.2)	0.20 (−0.04, 0.44)	0.24 (−0.20, 0.68)	0.04 (−0.39, 0.47)
**Situation at the end of the pathway**	Unchanged care or support level	162 (55.1)	70 (59.3)	78 (51.3)	14 (58.3)	0.25 (0.01, 0.49)	0.21 (−0.23, 0.65)	0.15 (−0.28, 0.58)
	New or increased home-based support or care	88 (29.9)	36 (30.5)	46 (30.3)	6 (25.0)			
	New institutional care	12 (4.1)	3 (2.5)	8 (5.3)	1 (4.2)			
	Other: Deceased or lost to follow-up	32 (10.9)	9 (7.6)	20 (13.2)	3 (12.5)			

^1^ Median [Q1, Q3]; *n* (%). ^2^ Pairwise Standardized Mean Difference (SMD) with 95% Confidence Interval (CI). ^3^ temp. = temporary; WBAT = weight-bearing as tolerated; MSK = musculoskeletal. ^4^ No SMD could be calculated due to the absence or few counts in certain categories. ^5^ The category of “*fracture or prosthetic-associated infections*” was omitted because there was no individual assigned to this category.

**Table 3:** Functional outcomes of the study cohort overall and stratified for each of the three hip fragility fracture groups. All variables were assessed at discharge from inpatient rehabilitation. The table includes only individuals who completed the inpatient rehabilitation program.

		Overall	FN-F	Tr-F	Ac/Pp-F	SMD (95% CI) ^2^		
		N = 255 (%) ^1^	N = 109 (%) ^1^	N = 126 (%) ^1^	N = 20 (%) ^1^	FN-F vs. Tr-F	FN-F vs. Ac/Pp-F	Tr-F vs. Ac/Pp-F
***Functional Independence Measure (FIM) at discharge of inpatient rehabilitation***								
**FIM—Total Score**		110.0 [103.0, 115.0]	111.0 [104.0, 117.0]	110.0 [103.0, 114.0]	107.5 [97.0, 113.0]	0.09 (−0.17, 0.34)	0.48 (−0.00, 0.96)	0.44 (−0.03, 0.91)
**FIM—Motor Sub-Score**		79.0 [73.0, 82.0]	80.0 [74.0, 83.0]	78.0 [72.0, 82.0]	76.0 [67.0, 80.0]	0.10 (−0.16, 0.36)	0.46 (−0.02, 0.94)	0.40 (−0.07, 0.88)
**FIM—Cognition Sub-Score**		32.0 [30.0, 34.0]	32.0 [30.0, 34.0]	32.0 [30.0, 34.0]	31.0 [28.0, 33.0]	0.02 (−0.24, 0.28)	0.39 (−0.09, 0.86)	0.42 (−0.06, 0.89)
***Timed Up and Go (TUG) test at discharge of inpatient rehabilitation***								
**TUG test time** (in seconds)		22.0 [17.0, 30.0]	20.0 [16.0, 25.5]	22.0 [17.0, 32.0]	27.0 [17.5, 34.5]	0.24 (−0.03, 0.51)	0.39 (−0.14, 0.92)	0.22 (−0.30, 0.75)
*Unknown*		*26*	*9*	*13*	*4*			
**Supportive device used during TUG test**	No gait device used	3 (1.3)	1 (1.0)	2 (1.8)	0 (0.0)	0.43 (0.16, 0.70)	0.38 (−0.15, 0.91)	0.58 (0.05, 1.10)
	Used 1–2 walking sticks	3 (1.3)	1 (1.0)	2 (1.8)	0 (0.0)			
	Used 1–2 crutches	130 (56.8)	65 (65.0)	56 (49.6)	9 (56.3)			
	Used rollator	79 (34.5)	31 (31.0)	41 (36.3)	7 (43.8)			
	Used walking frame	14 (6.1)	2 (2.0)	12 (10.6)	0 (0.0)			
	*Unknown*	*26*	*9*	*13*	*4*			

^1^ Median [Q1, Q3]; *n* (%). ^2^ Pairwise Standardized Mean Difference (SMD) with 95% Confidence Interval (CI).

## Data Availability

The data presented in this study cannot be shared due to restrictions imposed by Swiss data protection legislation, namely the Swiss Federal Act on Data Protection. Under this act, the data are classified as highly sensitive personal health data, forbidden to share.

## Abbreviations

The following abbreviations are used in this manuscript:

Ac/Pp-F	Acetabulum/periprosthetic fracture
CFS	Clinical Frailty Scale
CIRS	Cumulative Illness Rating Scale
FIM	Functional Independence Measure
FN-F	Femoral neck fracture
ICD	International Classification of Disease
LTHF	Low-trauma hip fracture
MSK	Musculoskeletal
SMD	Standardized Mean Difference
Tr-F	Trochanteric fracture
TUG	Timed Up and Go
WBAT	Weight-bearing as tolerated

## Appendix A

In Appendix A, additional variables are reported. Further description of the variables can be found in Appendix B.

**Table A1:** Pre-fracture place and situation of living.

		Overall	FN-F	Tr-F	Ac/Pp-F	SMD (95% CI) ^2^		
		N = 294 (%) ^1^	N = 118 (%) ^1^	N = 152 (%) ^1^	N = 24 (%) ^1^	FN-F vs. Tr-F	FN-F vs. Ac/Pp-F	Tr-F vs. Ac/Pp-F
**Marital status**	Single	30 (10.2)	11 (9.3)	15 (9.9)	4 (16.7)	0.11 (−0.13, 0.35)	0.70 (0.25, 1.14)	0.61 (0.18, 1.05)
	Married or registered partnership	121 (41.2)	44 (37.3)	64 (42.1)	13 (54.2)			
	Divorced from marriage or registered partnership	34 (11.6)	14 (11.9)	16 (10.5)	4 (16.7)			
	Widowed	109 (37.1)	49 (41.5)	57 (37.5)	3 (12.5)			
**Living situation**	Living alone	122 (41.5)	50 (42.4)	64 (42.1)	8 (33.3)	0.10 (−0.14, 0.34)	0.30 (−0.14, 0.74)	0.33 (−0.10, 0.77)
	Living with partner	121 (41.2)	47 (39.8)	61 (40.1)	13 (54.2)			
	Living with or close by relatives or people close to the person	41 (13.9)	16 (13.6)	23 (15.1)	2 (8.3)			
	Living in a social medical institution	10 (3.4)	5 (4.2)	4 (2.6)	1 (4.2)			
**Preparation of medication at living place**	Self/independent	232 (79.7)	100 (85.5)	116 (77.3)	16 (66.7)	0.23 (−0.01, 0.47)	0.48 (0.03, 0.92)	0.39 (−0.05, 0.82)
	Non-healthcare professional (e.g., relative or friend)	27 (9.3)	7 (6.0)	18 (12.0)	2 (8.3)			
	Healthcare professional including pharmacists	32 (11.0)	10 (8.5)	16 (10.7)	6 (25.0)			
	*Unknown*	*3*	*1*	*2*	*0*			
**Patient-reported sleep** **information**	No sleep problems	165 (56.1)	64 (54.2)	90 (59.2)	11 (45.8)	0.33 (0.09, 0.58)	0.55 (0.11, 0.99)	0.71 (0.28, 1.15)
	Sometimes or mild sleep problems	28 (9.5)	11 (9.3)	15 (9.9)	2 (8.3)			
	Permanent or severe sleep problems	62 (21.1)	26 (22.0)	31 (20.4)	5 (20.8)			
	Taking sleep medications	7 (2.4)	2 (1.7)	5 (3.3)	0 (0.0)			
	Taking Z-drugs (sedative-hypnotics)	21 (7.1)	10 (8.5)	8 (5.3)	3 (12.5)			
	Taking other medications for sleep (e.g., analgesics)	2 (0.7)	2 (1.7)	0 (0.0)	0 (0.0)			
	CPAP/BiPAP, O2-Mask	5 (1.7)	2 (1.7)	0 (0.0)	3 (12.5)			
	*Unknown*	*4*	*1*	*3*	*0*			
**Patient-reported** **Smoking status**	Never	34 (23.4)	9 (19.1)	21 (24.7)	4 (30.8)	0.27 (−0.09, 0.62)	0.33 (−0.29, 0.94)	0.14 (−0.44, 0.73)
	Former smoking, smoking stop ≥3 months ago	64 (44.1)	25 (53.2)	34 (40.0)	5 (38.5)			
	Currently smoking	47 (32.4)	13 (27.7)	30 (35.3)	4 (30.8)			
	*Unknown*	*149*	*71*	*67*	*11*			
**Patient-reported alcohol consumption**	No alcohol consumption	105 (41.0)	47 (44.8)	49 (36.8)	9 (50.0)	0.25 (−0.00, 0.51)	0.26 (−0.24, 0.76)	0.27 (−0.22, 0.77)
	Mild-moderate	112 (43.8)	47 (44.8)	59 (44.4)	6 (33.3)			
	Abusive alcohol consumption	39 (15.2)	11 (10.5)	25 (18.8)	3 (16.7)			
	*Unknown*	*38*	*13*	*19*	*6*			

^1^ *n* (%). ^2^ Pairwise Standardized Mean Difference (SMD) with 95% Confidence Interval (CI).

**Table A2:** Pre-fracture walking ability, usage of assistive gait devices, and previous falls.

		Overall	FN-F	Tr-F	Ac/Pp-F	SMD (95% CI) ^2^		
		N = 294 (%) ^1^	N = 118 (%) ^1^	N = 152 (%) ^1^	N = 24 (%) ^1^	FN-F vs. Tr-F	FN-F vs. Ac/Pp-F	Tr-F vs. Ac/Pp-F
**Walking ability** ^3^	Independent, without assistance	161 (54.8)	66 (55.9)	83 (54.6)	12 (50.0)	0.07 (−0.17, 0.31)	0.51 (0.07, 0.95)	0.46 (0.03, 0.90)
	Independent indoors, assistance only outdoor with 1 walking stick	43 (14.6)	15 (12.7)	23 (15.1)	5 (20.8)			
	Independent indoors, assistance only outdoor with 2 walking stick or rollator	80 (27.2)	34 (28.8)	42 (27.6)	4 (16.7)			
	Somewhat independent indoors, assistance needed from a person outdoors	10 (3.4)	3 (2.5)	4 (2.6)	3 (12.5)			
**No gait device used at all or only for short distances**		230 (78.2)	92 (78.0)	119 (78.3)	19 (79.2)	0.01 (−0.23, 0.25)	0.03 (−0.41, 0.47)	0.02 (−0.41, 0.45)
**Using 1–2 walking sticks**		54 (18.4)	19 (16.1)	31 (20.4)	4 (16.7)	0.11 (−0.13, 0.35)	0.02 (−0.42, 0.45)	0.10 (−0.33, 0.53)
**Using 1–2 crutches**		26 (8.8)	13 (11.0)	10 (6.6)	3 (12.5)	0.16 (−0.08, 0.40)	0.05 (−0.39, 0.48)	0.20 (−0.23, 0.63)
**Using rollator**		65 (22.1)	26 (22.0)	34 (22.4)	5 (20.8)	0.01 (−0.23, 0.25)	0.03 (−0.41, 0.47)	0.04 (−0.39, 0.47)
**Using wheelchair**		4 (1.4)	2 (1.7)	0 (0.0)	2 (8.3)	0.19 (−0.06, 0.43)	0.31 (−0.13, 0.75)	0.43 (−0.01, 0.86)

^1^ *n* (%). ^2^ Pairwise Standardized Mean Difference (SMD) with 95% Confidence Interval (CI). ^3^ Note for the walking ability, the category of *no functional walking ability* was left away because there was no individual assigned to this category.

**Table A3:** Severity of illness and presence of comorbidities.

	Overall	FN-F	Tr-F	Ac/Pp-F	SMD (95% CI) ^2^		
	N = 294 (%) ^1^	N = 118 (%) ^1^	N = 152 (%) ^1^	N = 24 (%) ^1^	FN-F vs. Tr-F	FN-F vs. Ac/Pp-F	Tr-F vs. Ac/Pp-F
**Experienced previous low-trauma fracture(s) at other** **locations than the hip, pelvis, or femur** *—unknown*	80 (27.7) *5*	23 (20.0) *3*	49 (32.7) *2*	8 (33.3)*0*	0.29 (0.05, 0.53)	0.3 (−0.14, 0.75)	0.01 (−0.42, 0.45)
**Osteoporosis**	109 (37.1)	38 (32.2)	66 (43.4)	5 (20.8)	0.23 (−0.01, 0.47)	0.26 (−0.18, 0.70)	0.50 (0.06, 0.93)
**Osteoarthritis and other arthropathies**	102 (34.7)	37 (31.4)	59 (38.8)	6 (25.0)	0.16 (−0.08, 0.40)	0.14 (−0.30, 0.58)	0.30 (−0.13, 0.73)
**Other diseases of the musculoskeletal system, and soft and** **connective tissue**	48 (16.3)	21 (17.8)	24 (15.8)	3 (12.5)	0.05 (−0.19, 0.29)	0.15 (−0.29, 0.59)	0.09 (−0.34, 0.53)
**Spondylopathies and other spine related diseases**	99 (33.7)	29 (24.6)	63 (41.4)	7 (29.2)	0.36 (0.12, 0.61)	0.10 (−0.34, 0.54)	0.26 (−0.17, 0.69)
**Mild cognitive impairment, early stage of/highly suspected** **dementia, and/or other cognitive difficulties**	31 (10.5)	11 (9.3)	16 (10.5)	4 (16.7)	0.04 (−0.20, 0.28)	0.22 (−0.22, 0.66)	0.18 (−0.25, 0.61)
**Dementia**	16 (5.4)	8 (6.8)	6 (3.9)	2 (8.3)	0.13 (−0.11, 0.37)	0.06 (−0.38, 0.50)	0.18 (−0.25, 0.61)
**Substance-use disorders**	42 (14.3)	14 (11.9)	23 (15.1)	5 (20.8)	0.10 (−0.14, 0.34)	0.24 (−0.20, 0.68)	0.15 (−0.28, 0.58)
**Other psychiatric disease and mental health diseases**	62 (21.1)	22 (18.6)	31 (20.4)	9 (37.5)	0.04 (−0.20, 0.28)	0.43 (−0.01, 0.87)	0.38 (−0.05, 0.82)
**Polyneuropathies**	27 (9.2)	6 (5.1)	20 (13.2)	1 (4.2)	0.28 (0.04, 0.52)	0.04 (−0.40, 0.48)	0.32 (−0.11, 0.76)
**Parkinson’s, multiple sclerosis, restless leg syndrome, and** **other extra pyramidal diseases**	27 (9.2)	12 (10.2)	12 (7.9)	3 (12.5)	0.08 (−0.16, 0.32)	0.07 (−0.37, 0.51)	0.15 (−0.28, 0.58)
**Diabetes mellitus**	48 (16.3)	17 (14.4)	24 (15.8)	7 (29.2)	0.04 (−0.20, 0.28)	0.36 (−0.08, 0.80)	0.32 (−0.11, 0.76)
**Thyroid and parathyroid diseases**	65 (22.1)	22 (18.6)	39 (25.7)	4 (16.7)	0.17 (−0.07, 0.41)	0.05 (−0.39, 0.49)	0.22 (−0.21, 0.65)
**Vitamin D and folic acid deficiencies**	197 (67.0)	81 (68.6)	102 (67.1)	14 (58.3)	0.03 (−0.21, 0.27)	0.22 (−0.22, 0.65)	0.18 (−0.25, 0.61)
**Malnutrition and other anemia**	125 (42.5)	42 (35.6)	69 (45.4)	14 (58.3)	0.2 (−0.04, 0.44)	0.47 (0.03, 0.91)	0.26 (−0.17, 0.69)
**Adiposities and overnutrition**	43 (14.6)	13 (11.0)	27 (17.8)	3 (12.5)	0.19 (−0.05, 0.43)	0.05 (−0.39, 0.48)	0.15 (−0.28, 0.58)
**Lipidemia including hyperlipidemia**	96 (32.7)	34 (28.8)	53 (34.9)	9 (37.5)	0.13 (−0.11, 0.37)	0.19 (−0.25, 0.62)	0.05 (−0.38, 0.49)
**Other metabolic and nutritional disruption**	69 (23.5)	26 (22.0)	37 (24.3)	6 (25.0)	0.05 (−0.19, 0.30)	0.07 (−0.37, 0.51)	0.02 (−0.42, 0.45)
**Coronary heart diseases**	62 (21.1)	24 (20.3)	33 (21.7)	5 (20.8)	0.03 (−0.21, 0.27)	0.01 (−0.43, 0.45)	0.02 (−0.41, 0.45)
**Congestive heart failure**	43 (14.6)	19 (16.1)	22 (14.5)	2 (8.3)	0.05 (−0.20, 0.29)	0.24 (−0.20, 0.68)	0.19 (−0.24, 0.63)
**Arrhythmia and conduction disorders**	87 (29.6)	41 (34.7)	41 (27.0)	5 (20.8)	0.17 (−0.07, 0.41)	0.31 (−0.13, 0.75)	0.14 (−0.29, 0.58)
**Other ischemic heart and other forms of heart disease**	59 (20.1)	21 (17.8)	35 (23.0)	3 (12.5)	0.13 (−0.11, 0.37)	0.15 (−0.29, 0.59)	0.28 (−0.15, 0.71)
**Hypertensive diseases**	210 (71.4)	74 (62.7)	120 (78.9)	16 (66.7)	0.36 (0.12, 0.61)	0.08 (−0.36, 0.52)	0.28 (−0.15, 0.71)
**Arterial diseases and other cardiovascular**	81 (27.6)	27 (22.9)	47 (30.9)	7 (29.2)	0.18 (−0.06, 0.42)	0.14 (−0.30, 0.58)	0.04 (−0.39, 0.47)
**Chronic obstructive pulmonary disease**	37 (12.6)	13 (11.0)	21 (13.8)	3 (12.5)	0.08 (−0.16, 0.33)	0.05 (−0.39, 0.48)	0.04 (−0.39, 0.47)
**Renal insufficiency**	110 (37.4)	43 (36.4)	58 (38.2)	9 (37.5)	0.04 (−0.20, 0.28)	0.02 (−0.42, 0.46)	0.01 (−0.42, 0.44)
**Malignant tumor without metastasis**	58 (19.7)	20 (16.9)	34 (22.4)	4 (16.7)	0.14 (−0.10, 0.38)	0.01 (−0.43, 0.45)	0.14 (−0.29, 0.58)
**Malignant tumor with metastasis**	5 (1.7)	1 (0.8)	2 (1.3)	2 (8.3)	0.05 (−0.20, 0.29)	0.36 (−0.08, 0.80)	0.33 (−0.10, 0.76)

^1^ *n* (%). ^2^ Pairwise Standardized Mean Difference (SMD) with 95% Confidence Interval (CI).

**Table A4:** Descriptives from the acute care setting.

		Overall	FN-F	Tr-F	Ac/Pp-F	SMD (95% CI) ^2^		
		N = 294 (%) ^1^	N = 118 (%) ^1^	N = 152 (%) ^1^	N = 24 (%) ^1^	FN-F vs. Tr-F	FN-F vs. Ac/Pp-F	Tr-F vs. Ac/Pp-F
**Health insurance**	Compulsory	228 (77.6)	92 (78.0)	118 (77.6)	18 (75.0)	0.18 (−0.06, 0.43)	0.10 (−0.33, 0.54)	0.13 (−0.30, 0.56)
	Additional half private	56 (19.0)	20 (16.9)	31 (20.4)	5 (20.8)			
	Additional private	10 (3.4)	6 (5.1)	3 (2.0)	1 (4.2)			
**Length of first stay in acute care hospital** (in days)		9.5 [8.0, 13.0]	9.0 [7.0, 12.0]	10.0 [8.0, 13.0]	11.5 [8.5, 14.0]	0.19 (−0.06, 0.43)	0.32 (−0.12, 0.76)	0.01 (−0.42, 0.44)
**American Society of** **Anesthesiologists** **Grading (ASA)** ^3^	ASA Grade II	106 (36.1)	46 (39.0)	50 (32.9)	10 (41.7)	0.23 (−0.02, 0.47)	0.15 (−0.29, 0.59)	0.19 (−0.24, 0.62)
	ASA Grade III	172 (58.5)	63 (53.4)	96 (63.2)	13 (54.2)			
	ASA Grade IV	16 (5.4)	9 (7.6)	6 (3.9)	1 (4.2)			
**Number of acute care stays** (over the entire pathway)	One stay	250 (85.0)	99 (83.9)	129 (84.9)	22 (91.7)	0.31 (0.07, 0.55)	0.29 (−0.15, 0.73)	0.47 (0.04, 0.91)
	Two stays	38 (12.9)	14 (11.9)	23 (15.1)	1 (4.2)			
	Three or more stays	6 (2.0)	5 (4.2)	0 (0.0)	1 (4.2)			

^1^ Median [Q1, Q3]; *n* (%). ^2^ Pairwise Standardized Mean Difference (SMD) with 95% Confidence Interval (CI). ^3^ The category *ASA Grade I* and *Grade V* was left away because there was no individual assigned to this category.

**Table A5:** Descriptives from the acute care setting from participants who received surgery.

		Overall	FN-F	Tr-F	Ac/Pp-F	SMD (95% CI) ^2^		
		N = 281 (%) ^1^	N = 114 (%) ^1^	N = 149 (%) ^1^	N = 18 (%) ^1^	FN-F vs. Tr-F	FN-F vs. Ac/Pp-F	Tr-F vs. Ac/Pp-F
**Time from fracture to surgery** (in days)		1.00 [1.0, 2.0]	2.0 [1.0, 4.0]	1.0 [0.0, 2.0]	3.0 [1.0, 4.0]	0.47 (0.22, 0.72)	0.17 (−0.33, 0.67)	0.62 (0.13, 1.12)
**Surgery instance**	1st surgery	266 (94.7)	114 (100.0)	149 (100.0)	3 (16.7)	0.0 (−0.24, 0.24)	3.16 (2.54, 3.79)	3.16 (2.57, 3.76)
	1st revision (2nd surgery)	11 (3.9)	0 (0.0)	0 (0.0)	11 (61.1)			
	2nd revision (3rd surgery)	4 (1.4)	0 (0.0)	0 (0.0)	4 (22.2)			
**Type of surgery**	Dynamic Hip Screws	8 (2.8)	2 (1.8)	6 (4.0)	0 (0.0)	4.68 (4.21, 5.15)	10.58 (9.21, 11.95)	12.12 (10.74, 13.51)
	Nail	139 (49.5)	3 (2.6)	136 (91.3)	0 (0.0)			
	Hemiarthroplasty	34 (12.1)	34 (29.8)	0 (0.0)	0 (0.0)			
	Total hip arthroplasty	78 (27.8)	73 (64.0)	5 (3.4)	0 (0.0)			
	Other	22 (7.8)	2 (1.8)	2 (1.3)	18 (100.0)			
**Change of implant** ^3^		8 (2.8)	-	-	8 (44.4)			
**Additional osteosynthesis** ^3^		12 (4.3)	-	-	12 (66.7)			
**Clavien–Dindo** **post-surgical complications classification** ^4^	No Deviations from the normal post-operative course	97 (34.9)	54 (47.4)	39 (26.7)	4 (22.2)	0.54 (0.29, 0.79)	0.57 (0.07, 1.07)	0.34 (−0.15, 0.83)
	Grade I	35 (12.6)	14 (12.3)	18 (12.3)	3 (16.7)			
	Grade II	48 (17.3)	19 (16.7)	24 (16.4)	5 (27.8)			
	Grade III	81 (29.1)	20 (17.5)	56 (38.4)	5 (27.8)			
	Grade IV	17 (6.1)	7 (6.1)	9 (6.2)	1 (5.6)			
	*Unknown*	*3*	*0*	*3*	*0*			
**Number of additional** **surgeries** (over the entire pathway)	Zero	269 (95.7)	110 (96.5)	142 (95.3)	17 (94.4)	0.08 (−0.16, 0.32)	0.20 (−0.30, 0.70)	0.14 (−0.35, 0.63)
	One	10 (3.6)	3 (2.6)	6 (4.0)	1 (5.6)			
	Two or more	2 (0.7)	1 (0.9)	1 (0.7)	0 (0.0)			
**Reason for re-op** ^5^	Periprosthetic fracture	4 (33.3)	1 (25.0)	3 (42.9)	0 (0.0)			
	Failure of osteosynthesis or endoprosthesis	1 (8.3)	0 (0.0)	1 (14.3)	0 (0.0)			
	Infection	2 (16.7)	1 (25.0)	1 (14.3)	0 (0.0)			
	Hematoma or seroma	3 (25.0)	2 (50.0)	1 (14.3)	0 (0.0)			
	Other	2 (16.7)	0 (0.0)	1 (14.3)	1 (100.0)			
**Main measure taken** **for re-op** ^5^	Change of implant	0 (0.0)	0 (0.0)	0 (0.0)	0 (0.0)			
	Conversion to total hip arthroplasty	5 (41.7)	0 (0.0)	5 (71.4)	0 (0.0)			
	Partial change of prothesis	4 (33.3)	3 (75.0)	0 (0.0)	1 (100.0)			
	Removal of implant or osteosynthesis material	0 (0.0)	0 (0.0)	0 (0.0)	0 (0.0)			
	Rinsing or debridement	2 (16.7)	0 (0.0)	2 (28.6)	0 (0.0)			
	Other	1 (8.3)	1 (25.0)	0 (0.0)	0 (0.0)			
**Reason for 2nd re-op** ^5^	Infection	1 (0.4)	0 (0.0)	1 (0.7)	0 (0.0)			
**Main measure taken** **for 2nd re-op** ^5^	Rinsing or debridement	1 (0.4)	0 (0.0)	1 (0.7)	0 (0.0)			

^1^ Median [Q1, Q3]; *n* (%). ^2^ Pairwise Standardized Mean Difference (SMD) with 95% Confidence Interval (CI). ^3^ Only for periprosthetic fractures. ^4^ The category *Grade V* was left away because there was no individual assigned to this category. ^5^ No SMD could be calculated due to the absent or few counts in certain categories.

**Table A6:** Descriptives from the rehabilitation setting.

		Overall	FN-F	Tr-F	Ac/Pp-F	SMD (95% CI) ^2^		
		N = 294 (%) ^1^	N = 118 (%) ^1^	N = 152 (%) ^1^	N = 24 (%) ^1^	FN-F vs. Tr-F	FN-F vs. Ac/Pp-F	Tr-F vs. Ac/Pp-F
**Number of medications**		4.0 [3.0, 6.0]	5.0 (3.0, 6.0]	4.0 (3.0, 7.0]	4.5 (3.5, 6.5]	0.08 (−0.16, 0.32)	0.14 (−0.30, 0.57)	0.05 (−0.38, 0.48)
**Pain Numerical Rating Scale**		3.0 [2.0, 5.0]	3.00 (1.0, 5.0]	4.00 (2.0, 6.0]	2.0 (0.0, 4.0]	0.43 (0.18, 0.68)	0.26 (−0.19, 0.71)	0.70 (0.26, 1.15)
*Unknown*		*9*	*4*	*4*	*1*			
**Nutritional Risk Screening**		3.0 [2.0, 3.0]	2.0 [2.0, 3.0]	3.0 [2.0, 3.0]	2.5 [2.0, 3.5]	0.11 (−0.13, 0.35)	0.11 (−0.33, 0.55)	0.0 (−0.43, 0.43)
*Unknown*		*3*	*1*	*2*	*0*			
**Geriatric Depression Scale Brief Total Score** ^3,4^		1.0 [0.0, 1.0]	1.0 [0.0, 1.0]	1.0 [0.0, 2.0]	0.0 [0.0, 1.0]	0.06 (−0.56, 0.69)	0.77 (−0.50, 2.03)	0.81 (−0.40, 2.01)
*Unknown*		*248*	*103*	*124*	*21*			
**Geriatric Depression Scale Short Total Score** ^3,4^		3.0 [2.0, 5.0]	2.5 [2.0, 4.0]	3.0 [2.0, 5.0]	3.5 [0.0, 5.0]	0.25 (−0.27, 0.76)	0.27 (−0.62, 1.16)	0.08 (−0.79, 0.94)
*Unknown*		*228*	*92*	*118*	*18*			
**Mini Mental State Examen Total Score** ^3^		26.0 [23.0, 28.0]	26.0 [23.0, 28.0]	26.0 [24.0, 28.0]	25.5 [20.0, 28.0]	0.34 (−0.05, 0.73)	0.23 (−0.46, 0.92)	0.14 (−0.54, 0.81)
*Unknown*		*181*	*73*	*94*	*14*			
**Clock Test Total Score** ^3^		5.0 [3.0, 7.0]	5.0 [3.0, 6.0]	5.0 [2.0, 6.0]	6.0 [3.5, 7.0]	0.04 (−0.39, 0.47)	0.45 (−0.32, 1.22)	0.42 (−0.33, 1.18)
*Unknown*		*202*	*81*	*105*	*16*			
**Length of stay in rehabilitation** (in days)		21.0 [18.0, 28.0]	21.0 [18.0, 27.0]	21.0 [18.0, 27.0]	28.0 [21.0, 32.0]	0.09 (−0.15, 0.33)	0.80 (0.35, 1.24)	0.68 (0.25, 1.12)
**Number of rehabilitations stays** (over the entire pathway)	One stay	273 (92.9)	106 (89.8)	145 (95.4)	22 (91.7)	0.21 (−0.03, 0.46)	0.06 (−0.38, 0.50)	0.15 (−0.28, 0.58)
	Two or more stays	21 (7.1)	12 (10.2)	7 (4.6)	2 (8.3)			
**Discontinuation of Rehabilitation**		52 (17.7)	16 (13.6)	32 (21.1)	4 (16.7)	0.20 (−0.04, 0.44)	0.09 (−0.35, 0.53)	0.11 (−0.32, 0.54)
	*No discontinuation*	*242*	*102*	*120*	*20*			
**Reason for discontinuation** **of rehabilitation**	Transfer to acute care hospital for longer than 24 h	31 (59.6)	11 (68.8)	19 (59.4)	1 (25.0)	0.56 (−0.05, 1.17)	1.18 (0.03, 2.34)	0.93 (−0.13, 1.99)
	Not possible to participate in rehabilitation	18 (34.6)	4 (25.0)	11 (34.4)	3 (75.0)			
	Other reasons	2 (3.8)	0 (0.0)	2 (6.3)	0 (0.0)			
	Death	1 (1.9)	1 (6.3)	0 (0.0)	0 (0.0)			
	*No discontinuation*	*242*	*102*	*120*	*20*			
**Returned to the rehabilitation facility after discontinuation**	Yes, and completed rehabilitation	12 (23.1)	6 (37.5)	6 (18.8)	0 (0.0)	0.43 (−0.18, 1.03)	1.1 (−0.05, 2.24)	0.68 (−0.37, 1.73)
	No, did not return	40 (76.9)	10 (62.5)	26 (81.3)	4 (100.0)			
	*No discontinuation*	*242*	*102*	*120*	*20*			
**Destination at the end of the pathway**	Independent living	123 (41.8)	58 (49.2)	59 (38.8)	6 (25.0)	0.37 (0.13, 0.61)	0.77 (0.32, 1.22)	0.49 (0.06, 0.93)
	Independent living with relatives	22 (7.5)	8 (6.8)	12 (7.9)	2 (8.3)			
	Independent living with care support	101 (34.4)	38 (32.2)	52 (34.2)	11 (45.8)			
	Temporary care home	17 (5.8)	2 (1.7)	12 (7.9)	3 (12.5)			
	Long-term care home	16 (5.4)	5 (4.2)	9 (5.9)	2 (8.3)			
	Acute care hospital	12 (4.1)	5 (4.2)	7 (4.6)	0 (0.0)			
	Other	1 (0.3)	1 (0.8)	0 (0.0)	0 (0.0)			
	Deceased	2 (0.7)	1 (0.8)	1 (0.7)	0 (0.0)			

^1^ Median (Q1, Q3); *n* (%). ^2^ Pairwise Standardized Mean Difference (SMD) with 95% Confidence Interval (CI). ^3^ Only participants, who underwent a specialized MSK–geriatric rehabilitation program or if clinically indicated, received these assessments (*Geriatric Depression Scale*, *Mini Mental State Examen*, and *Clock Test*). ^4^ Participants received either the brief (5 item) or short (15 item) version of the *Geriatric Depression Scale*.

**Table A7:** Functional Independence Measure with total scores and all 18 items at discharge of rehabilitation. The table shows only participants who completed the inpatient rehabilitation program.

		Overall	FN-F	Tr-F	Ac/Pp-F	SMD (95% CI) ^2^		
		N = 255 (%) ^1^	N = 109 (%) ^1^	N = 126 (%) ^1^	N = 20 (%) ^1^	FN-F vs. Tr-F	FN-F vs. Ac/Pp-F	Tr-F vs. Ac/Pp-F
**1. FIM item:** **Eating and drinking**	Total assistance	0 (0.0)	0 (0.0)	0 (0.0)	0 (0.0)	0.38 (0.13, 0.64)	0.43 (−0.05, 0.91)	0.14 (−0.33, 0.61)
	Maximal assistance	0 (0.0)	0 (0.0)	0 (0.0)	0 (0.0)			
	Moderate assistance	1 (0.4)	1 (0.9)	0 (0.0)	0 (0.0)			
	Minimal assistance	3 (1.2)	3 (2.8)	0 (0.0)	0 (0.0)			
	Supervision	6 (2.4)	2 (1.8)	3 (2.4)	1 (5.0)			
	Modified independence	64 (25.1)	20 (18.3)	38 (30.2)	6 (30.0)			
	Complete independence	181 (71.0)	83 (76.1)	85 (67.5)	13 (65.0)			
**2. FIM item:** **Grooming/body care**	Total assistance	0 (0.0)	0 (0.0)	0 (0.0)	0 (0.0)	0.35 (0.09, 0.61)	0.58 (0.10, 1.06)	0.63 (0.16, 1.11)
	Maximal assistance	0 (0.0)	0 (0.0)	0 (0.0)	0 (0.0)			
	Moderate assistance	1 (0.4)	1 (0.9)	0 (0.0)	0 (0.0)			
	Minimal assistance	6 (2.4)	4 (3.7)	0 (0.0)	2 (10.0)			
	Supervision	12 (4.7)	5 (4.6)	7 (5.6)	0 (0.0)			
	Modified independence	49 (19.2)	16 (14.7)	27 (21.4)	6 (30.0)			
	Complete independence	187 (73.3)	83 (76.1)	92 (73.0)	12 (60.0)			
**3. FIM item:** **Bathing**	Total assistance	8 (3.1)	4 (3.7)	3 (2.4)	1 (5.0)	0.47 (0.21, 0.73)	0.68 (0.19, 1.16)	0.61 (0.14, 1.09)
	Maximal assistance	7 (2.7)	4 (3.7)	1 (0.8)	2 (10.0)			
	Moderate assistance	19 (7.5)	2 (1.8)	15 (11.9)	2 (10.0)			
	Minimal assistance	55 (21.6)	22 (20.2)	29 (23.0)	4 (20.0)			
	Supervision	49 (19.2)	25 (22.9)	23 (18.3)	1 (5.0)			
	Modified independence	91 (35.7)	40 (36.7)	43 (34.1)	8 (40.0)			
	Complete independence	26 (10.2)	12 (11.0)	12 (9.5)	2 (10.0)			
**4. FIM item:** **Dressing upper body**	Total assistance	1 (0.4)	1 (0.9)	0 (0.0)	0 (0.0)	0.25 (−0.01, 0.51)	0.55 (0.07, 1.03)	0.52 (0.04, 0.99)
	Maximal assistance	0 (0.0)	0 (0.0)	0 (0.0)	0 (0.0)			
	Moderate assistance	0 (0.0)	0 (0.0)	0 (0.0)	0 (0.0)			
	Minimal assistance	14 (5.5)	5 (4.6)	5 (4.0)	4 (20.0)			
	Supervision	9 (3.5)	2 (1.8)	6 (4.8)	1 (5.0)			
	Modified independence	62 (24.3)	24 (22.0)	34 (27.0)	4 (20.0)			
	Complete independence	169 (66.3)	77 (70.6)	81 (64.3)	11 (55.0)			
**5. FIM item:** **Dressing lower body**	Total assistance	5 (2.0)	2 (1.8)	1 (0.8)	2 (10.0)	0.16 (−0.10, 0.41)	0.83 (0.34, 1.31)	0.88 (0.40, 1.36)
	Maximal assistance	14 (5.5)	5 (4.6)	7 (5.6)	2 (10.0)			
	Moderate assistance	11 (4.3)	4 (3.7)	6 (4.8)	1 (5.0)			
	Minimal assistance	24 (9.4)	12 (11.0)	12 (9.5)	0 (0.0)			
	Supervision	23 (9.0)	9 (8.3)	14 (11.1)	0 (0.0)			
	Modified independence	139 (54.5)	60 (55.0)	66 (52.4)	13 (65.0)			
	Complete independence	39 (15.3)	17 (15.6)	20 (15.9)	2 (10.0)			
**6. FIM item:** **Toileting and intime hygiene**	Total assistance	2 (0.8)	2 (1.8)	0 (0.0)	0 (0.0)	0.35 (0.09, 0.61)	0.48 (−0.00, 0.96)	0.49 (0.01, 0.96)
	Maximal assistance	5 (2.0)	1 (0.9)	3 (2.4)	1 (5.0)			
	Moderate assistance	6 (2.4)	2 (1.8)	3 (2.4)	1 (5.0)			
	Minimal assistance	7 (2.7)	4 (3.7)	3 (2.4)	0 (0.0)			
	Supervision	11 (4.3)	7 (6.4)	2 (1.6)	2 (10.0)			
	Modified independence	89 (34.9)	37 (33.9)	46 (36.5)	6 (30.0)			
	Complete independence	135 (52.9)	56 (51.4)	69 (54.8)	10 (50.0)			
**7. FIM item:** **Bladder management**	Total assistance	8 (3.1)	5 (4.6)	2 (1.6)	1 (5.0)	0.34 (0.09, 0.60)	0.62 (0.13, 1.10)	0.67 (0.20, 1.15)
	Maximal assistance	1 (0.4)	0 (0.0)	0 (0.0)	1 (5.0)			
	Moderate assistance	4 (1.6)	0 (0.0)	3 (2.4)	1 (5.0)			
	Minimal assistance	6 (2.4)	3 (2.8)	1 (0.8)	2 (10.0)			
	Supervision	8 (3.1)	3 (2.8)	4 (3.2)	1 (5.0)			
	Modified independence	128 (50.2)	52 (47.7)	69 (54.8)	7 (35.0)			
	Complete independence	100 (39.2)	46 (42.2)	47 (37.3)	7 (35.0)			
**8. FIM item:** **Bowel management**	Total assistance	3 (1.2)	3 (2.8)	0 (0.0)	0 (0.0)	0.35 (0.09, 0.61)	0.77 (0.28, 1.26)	0.70 (0.22, 1.18)
	Maximal assistance	0 (0.0)	0 (0.0)	0 (0.0)	0 (0.0)			
	Moderate assistance	5 (2.0)	1 (0.9)	2 (1.6)	2 (10.0)			
	Minimal assistance	3 (1.2)	0 (0.0)	2 (1.6)	1 (5.0)			
	Supervision	7 (2.7)	2 (1.8)	3 (2.4)	2 (10.0)			
	Modified independence	116 (45.5)	47 (43.1)	64 (50.8)	5 (25.0)			
	Complete independence	121 (47.5)	56 (51.4)	55 (43.7)	10 (50.0)			
**9. FIM item:** **Transfer bed–chair/wheelchair**	Total assistance	0 (0.0)	0 (0.0)	0 (0.0)	0 (0.0)	0.27 (0.01, 0.53)	0.48 (−0.00, 0.96)	0.41 (−0.06, 0.89)
	Maximal assistance	1 (0.4)	1 (0.9)	0 (0.0)	0 (0.0)			
	Moderate assistance	4 (1.6)	2 (1.8)	1 (0.8)	1 (5.0)			
	Minimal assistance	7 (2.7)	1 (0.9)	5 (4.0)	1 (5.0)			
	Supervision	12 (4.7)	6 (5.5)	6 (4.8)	0 (0.0)			
	Modified independence	183 (71.8)	77 (70.6)	92 (73.0)	14 (70.0)			
	Complete independence	48 (18.8)	22 (20.2)	22 (17.5)	4 (20.0)			
**10. FIM item:** **Transfer toilet**	Total assistance	1 (0.4)	0 (0.0)	0 (0.0)	1 (5.0)	0.32 (0.06, 0.58)	0.60 (0.11, 1.08)	0.53 (0.05, 1.01)
	Maximal assistance	1 (0.4)	1 (0.9)	0 (0.0)	0 (0.0)			
	Moderate assistance	2 (0.8)	2 (1.8)	0 (0.0)	0 (0.0)			
	Minimal assistance	8 (3.1)	2 (1.8)	6 (4.8)	0 (0.0)			
	Supervision	8 (3.1)	5 (4.6)	3 (2.4)	0 (0.0)			
	Modified independence	183 (71.8)	75 (68.8)	92 (73.0)	16 (80.0)			
	Complete independence	52 (20.4)	24 (22.0)	25 (19.8)	3 (15.0)			
**11. FIM item:** **Transfer bath tub/shower**	Total assistance	21 (8.2)	9 (8.3)	10 (7.9)	2 (10.0)	0.39 (0.13, 0.65)	0.38 (−0.09, 0.86)	0.64 (0.16, 1.12)
	Maximal assistance	1 (0.4)	0 (0.0)	1 (0.8)	0 (0.0)			
	Moderate assistance	6 (2.4)	4 (3.7)	1 (0.8)	1 (5.0)			
	Minimal assistance	15 (5.9)	3 (2.8)	12 (9.5)	0 (0.0)			
	Supervision	47 (18.4)	20 (18.3)	21 (16.7)	6 (30.0)			
	Modified independence	138 (54.1)	59 (54.1)	70 (55.6)	9 (45.0)			
	Complete independence	27 (10.6)	14 (12.8)	11 (8.7)	2 (10.0)			
**12. FIM item:** **Walking/wheelchair**	Total assistance	9 (3.5)	5 (4.6)	2 (1.6)	2 (10.0)	0.48 (0.22, 0.74)	0.65 (0.17, 1.14)	0.47 (−0.00, 0.95)
	Maximal assistance	16 (6.3)	2 (1.8)	11 (8.7)	3 (15.0)			
	Moderate assistance	0 (0.0)	0 (0.0)	0 (0.0)	0 (0.0)			
	Minimal assistance	2 (0.8)	0 (0.0)	2 (1.6)	0 (0.0)			
	Supervision	18 (7.1)	4 (3.7)	12 (9.5)	2 (10.0)			
	Modified independence	210 (82.4)	98 (89.9)	99 (78.6)	13 (65.0)			
	Complete independence	0 (0.0)	0 (0.0)	0 (0.0)	0 (0.0)			
**13. FIM item:** **Stairs**	Total assistance	53 (20.8)	13 (11.9)	31 (24.6)	9 (45.0)	0.43 (0.17, 0.69)	0.85 (0.36, 1.34)	0.56 (0.08, 1.03)
	Maximal assistance	21 (8.2)	8 (7.3)	12 (9.5)	1 (5.0)			
	Moderate assistance	7 (2.7)	3 (2.8)	3 (2.4)	1 (5.0)			
	Minimal assistance	6 (2.4)	3 (2.8)	3 (2.4)	0 (0.0)			
	Supervision	62 (24.3)	25 (22.9)	34 (27.0)	3 (15.0)			
	Modified independence	106 (41.6)	57 (52.3)	43 (34.1)	6 (30.0)			
	Complete independence	0 (0.0)	0 (0.0)	0 (0.0)	0 (0.0)			
**14. FIM item:** **Comprehension**	Total assistance	1 (0.4)	1 (0.9)	0 (0.0)	0 (0.0)	0.35 (0.10, 0.61)	0.63 (0.15, 1.11)	0.35 (−0.12, 0.83)
	Maximal assistance	2 (0.8)	1 (0.9)	1 (0.8)	0 (0.0)			
	Moderate assistance	2 (0.8)	1 (0.9)	1 (0.8)	0 (0.0)			
	Minimal assistance	9 (3.5)	5 (4.6)	3 (2.4)	1 (5.0)			
	Supervision	15 (5.9)	4 (3.7)	9 (7.1)	2 (10.0)			
	Modified independence	120 (47.1)	43 (39.4)	65 (51.6)	12 (60.0)			
	Complete independence	106 (41.6)	54 (49.5)	47 (37.3)	5 (25.0)			
**15. FIM item:** **Expression**	Total assistance	1 (0.4)	1 (0.9)	0 (0.0)	0 (0.0)	0.34 (0.08, 0.59)	0.60 (0.12, 1.08)	0.42 (−0.06, 0.89)
	Maximal assistance	1 (0.4)	0 (0.0)	1 (0.8)	0 (0.0)			
	Moderate assistance	2 (0.8)	1 (0.9)	1 (0.8)	0 (0.0)			
	Minimal assistance	4 (1.6)	3 (2.8)	1 (0.8)	0 (0.0)			
	Supervision	13 (5.1)	2 (1.8)	8 (6.3)	3 (15.0)			
	Modified independence	76 (29.8)	34 (31.2)	35 (27.8)	7 (35.0)			
	Complete independence	158 (62.0)	68 (62.4)	80 (63.5)	10 (50.0)			
**16. FIM item:** **Social interaction**	Total assistance	0 (0.0)	0 (0.0)	0 (0.0)	0 (0.0)	0.14 (−0.12, 0.40)	0.58 (0.09, 1.06)	0.55 (0.08, 1.03)
	Maximal assistance	0 (0.0)	0 (0.0)	0 (0.0)	0 (0.0)			
	Moderate assistance	1 (0.4)	1 (0.9)	0 (0.0)	0 (0.0)			
	Minimal assistance	2 (0.8)	0 (0.0)	0 (0.0)	2 (10.0)			
	Supervision	18 (7.1)	7 (6.4)	9 (7.1)	2 (10.0)			
	Modified independence	76 (29.8)	32 (29.4)	37 (29.4)	7 (35.0)			
	Complete independence	158 (62.0)	69 (63.3)	80 (63.5)	9 (45.0)			
**17. FIM item:** **Problem solving**	Total assistance	0 (0.0)	0 (0.0)	0 (0.0)	0 (0.0)	0.18 (−0.08, 0.43)	0.55 (0.07, 1.03)	0.49 (0.02, 0.97)
	Maximal assistance	1 (0.4)	1 (0.9)	0 (0.0)	0 (0.0)			
	Moderate assistance	3 (1.2)	1 (0.9)	1 (0.8)	1 (5.0)			
	Minimal assistance	7 (2.7)	2 (1.8)	3 (2.4)	2 (10.0)			
	Supervision	46 (18.0)	18 (16.5)	23 (18.3)	5 (25.0)			
	Modified independence	119 (46.7)	50 (45.9)	62 (49.2)	7 (35.0)			
	Complete independence	79 (31.0)	37 (33.9)	37 (29.4)	5 (25.0)			
**18. FIM item:** **Memory**	Total assistance	0 (0.0)	0 (0.0)	0 (0.0)	0 (0.0)	0.24 (−0.02, 0.49)	0.36 (−0.12, 0.84)	0.34 (−0.13, 0.82)
	Maximal assistance	2 (0.8)	1 (0.9)	0 (0.0)	1 (5.0)			
	Moderate assistance	1 (0.4)	1 (0.9)	0 (0.0)	0 (0.0)			
	Minimal assistance	8 (3.1)	2 (1.8)	5 (4.0)	1 (5.0)			
	Supervision	25 (9.8)	10 (9.2)	13 (10.3)	2 (10.0)			
	Modified independence	98 (38.4)	41 (37.6)	49 (38.9)	8 (40.0)			
	Complete independence	121 (47.5)	54 (49.5)	59 (46.8)	8 (40.0)			

^1^ *n* (%). ^2^ Pairwise Standardized Mean Difference (SMD) with 95% Confidence Interval (CI)

**Table A8:** Separate selected key descriptives for the acetabular and periprosthetic fractures, which together form the heterogenous Ac/Pp-F group.

		Acetabular Fractures	Periprosthetic Fractures
		N = 6 (%) ^1^	N = 18 (%) ^1^
*Baseline Characteristics*			
**Age**		76.7 ± 8.6	80.4 ± 7.7
**Sex**	Female	2 (33.3)	12 (66.7)
	Male	4 (66.7)	6 (33.3)
**Clinical Frailty Scale ^2^**	1—Very fit	0 (0.0)	1 (5.6)
	2—Well	2 (33.3)	1 (5.6)
	3—Managing well	0 (0.0)	3 (16.7)
	4—Vulnerable	1 (16.7)	4 (22.2)
	5—Mildly frail	1 (16.7)	5 (27.8)
	6—Moderately frail	1 (16.7)	1 (5.6)
	7—Severely frail	1 (16.7)	3 (16.7)
**Presence of previous falls**		3 (50.0)	8 (44.4)
**Experience of gait and balance disturbances**		2 (33.3)	2 (11.1)
**Cumulative Illness Rating Scale—Total Score**		20.3 ± 4.4	20.9 ± 4.5
*Pathway characteristics*			
**Pathway Type**	P1—Acute care to rehabilitation	3 (50.0)	7 (38.9)
	P2—Acute care to temp. ^3^ care home to rehabilitation	3 (50.0)	7 (38.9)
	P3—Acute care to rehabilitation to temp. ^3^ care home	0 (0.0)	2 (11.1)
	P4—All with re-hospitalization	0 (0.0)	2 (11.1)
**Length of Pathway** (from fracture to pathway destination in days)		68.00 [55.0, 80.0]	48.50 [34.0, 90.0]
**Acute Care Treatment Schema**	Directly surgery	4 (66.7)	14 (77.8)
	Initial conservative therapy, followed by surgery	0 (0.0)	0 (0.0)
	Only conservative therapy	2 (33.3)	4 (22.2)
**Length of first stay in acute care hospital** (in days)		10.0 [8.0, 11.0]	12.0 [9.0, 14.0]
**Prescribed weight-bearing**	No weight-bearing	3 (50.0)	4 (22.2)
	Partial weight-bearing	3 (50.0)	13 (72.2)
	Full weight-bearing or WBAT ^3^	0 (0.0)	1 (5.6)
**Rehabilitation discipline**	Standard MSK ^3^	4 (66.7)	10 (55.6)
	Specialized MSK ^3^–geriatric	2 (33.3)	8 (44.4)
**Length of stay in rehabilitation** (in days)		30.0 (25.0, 46.0)	28.0 (21.0, 31.0)
**Destination is a private living setting**		6 (100.0)	13 (72.2)
**Situation at the End of the** **Pathway**	Unchanged care or support level	3 (50.0)	11 (61.1)
	New or increased home-based support or care	3 (50.0)	3 (16.7)
	New institutional care	0 (0.0)	1 (5.6)
	Other: Deceased or lost to follow-up	0 (0.0)	3 (16.7)
*Surgery descriptives*		**Acetabular Fractures** **N = 4 (%)** ^1^	**Periprosthetic Fractures** **N = 14 (%)** ^1^
**Time from fracture to surgery** (in days)		3.5 [3.0, 4.0]	3.0 [1.0, 4.0]
**Surgery instance**	1st surgery	3 (75.0)	0 (0.0)
	1st revision (2nd surgery)	1 (25.0)	10 (71.4)
	2nd revision (3rd surgery)	0 (0.0)	4 (28.6)
*Functional outcomes at discharge of inpatient rehabilitation* *(only participants who completed the inpatient rehabilitation program)*		**Acetabular Fractures** **N = 6 (%)** ^1^	**Periprosthetic Fractures** **N = 14 (%)** ^1^
**FIM—Total Score**		100.0 [95.0, 113.0]	109.5 [102.0, 113.0]
**FIM—Motor Sub-Score**		69.0 [66.0, 80.0]	77.0 [72.0, 80.0]
**FIM—Cognition Sub-Score**		31.0 [29.0, 33.0]	31.0 [27.0, 33.0]
**TUG test time** (in seconds)		30.0 [11.0, 34.0]	26.0 [18.0, 35.0]
*Unknown*		*3*	*1*
**Supportive device used during TUG test**	No gait device used	0 (0.0%)	0 (0.0%)
	Used 1–2 walking sticks	0 (0.0%)	0 (0.0%)
	Used 1–2 crutches	2 (66.7%)	7 (53.8%)
	Used rollator	1 (33.3%)	6 (46.2%)
	Used walking frame	0 (0.0%)	0 (0.0%)
	*Unknown*	*3*	*1*

^1^ Mean ± SD; median [Q1, Q3]; *n* (%). ^2^ For the Clinical Frailty Scale (CFS), the last two levels (8—very severely frail; and 9—terminally ill) were left away because there was no individual assigned to these two categories. ^3^ temp. = temporary; WBAT = weight-bearing as tolerated; MSK = musculoskeletal.

**Table A9:** Standardized Mean Differences (SMDs) with 95% confidence intervals (CIs) for selected key characteristics, comparing the combined Ac/Pp-F group and periprosthetic fractures alone (without the acetabular fractures) with the FN-F and Tr-F groups, and absolute differences between the corresponding SMDs.

		SMD (95% CI) ^1^				Absolute Difference in SMD	
		FN-F ^2^ vs. Periprosthetic Fractures ^3^	Tr-F vs. ^4^ Periprosthetic Fractures ^3^	FN-F ^2^ vs. Ac/Pp-F ^5^	Tr-F vs. ^4^ Ac/Pp-F ^5^	|A − C|	|B − D|
		A	B	C	D		
*Baseline Characteristics*							
**Age**		0.06 (−0.44, 0.55)	0.15 (−0.34, 0.64)	0.18 (−0.26, 0.62)	0.26 (−0.17, 0.70)	0.12	0.11
**Sex**	Female	0.01 (−0.49, 0.50)	0.05 (−0.44, 0.53)	0.18 (−0.26, 0.62)	0.13 (−0.30, 0.56)	0.17	0.08
	Male						
**Clinical Frailty Scale ^3^**	1—Very fit	0.70 (0.19, 1.20)	0.81 (0.31, 1.31)	0.54 (0.10, 0.98)	0.68 (0.24, 1.12)	0.16	0.13
	2—Well						
	3—Managing well						
	4—Vulnerable						
	5—Mildly frail						
	6—Moderately frail						
	7—Severely frail						
**Presence of previous falls**		0.16 (−0.33, 0.66)	0.14 (−0.35, 0.63)	0.13 (−0.30, 0.57)	0.11 (−0.32, 0.54)	0.03	0.03
**Experience of gait and balance disturbances**		0.23 (−0.26, 0.73)	0.17 (−0.32, 0.66)	0.07 (−0.37, 0.51)	0.01 (−0.42, 0.44)	0.16	0.16
**Cumulative Illness Rating Scale—Total Score**		0.13 (−0.37, 0.63)	0.05 (−0.44, 0.53)	0.10 (−0.34, 0.54)	0.08 (−0.35, 0.51)	0.03	0.03
*Pathway characteristics*							
**Pathway type**	P1—Acute care to rehabilitation	1.25 (0.74, 1.77)	0.81 (0.31, 1.31)	1.25 (0.79, 1.71)	0.84 (0.40, 1.28)	0.0	0.03
	P2—Acute care to temp. ^4^ care home to rehabilitation						
	P3—Acute care to rehabilitation to temp. ^4^ care home						
	P4—All with re-hospitalization						
**Length of pathway** (from fracture to pathway destination in days)		1.06 (0.54, 1.57)	0.62 (0.13, 1.11)	1.2 (0.74, 1.66)	0.69 (0.26, 1.13)	0.14	0.07
**Acute Care Treatment Schema**	Directly surgery	0.72 (0.22, 1.22)	0.66 (0.17, 1.16)	0.77 (0.32, 1.22)	0.72 (0.29, 1.16)	0.05	0.06
	Initial conservative therapy followed by surgery						
	Only conservative therapy						
**Length of first stay in acute care hospital** (in days)		0.41 (−0.08, 0.91)	0.07 (−0.42, 0.56)	0.32 (−0.12, 0.76)	0.01 (−0.42, 0.44)	0.09	0.06
**Prescribed weight-bearing**	No weight-bearing	2.51 (1.94, 3.09)	1.67 (1.15, 2.19)	2.67 (2.13, 3.21)	1.75 (1.29, 2.22)	0.16	0.08
	Partial weight-bearing						
	Full weight-bearing or WBAT ^4^						
**Rehabilitation discipline**	Standard MSK ^4^	0.06 (−0.44, 0.56)	0.05 (−0.44, 0.54)	0.0 (−0.44, 0.44)	0.01 (−0.42, 0.44)	0.06	0.04
	Specialized MSK ^4^–geriatric						
**Length of stay in rehabilitation** (in days)		0.64 (0.14, 1.15)	0.52 (0.03, 1.02)	0.80 (0.35, 1.24)	0.68 (0.25, 1.12)	0.16	0.16
**Destination is a private living setting**		0.41 (−0.09, 0.91)	0.21 (−0.28, 0.70)	0.24 (−0.20, 0.68)	0.04 (−0.39, 0.47)	0.17	0.17
**Situation at the end of the pathway**	Unchanged care or support level	0.42 (−0.08, 0.92)	0.33 (−0.16, 0.82)	0.21 (−0.23, 0.65)	0.15 (−0.28, 0.58)	0.21	0.18
	New or increased home-based support or care						
	New institutional care						
	Other: Deceased or lost to follow-up						
*Surgery descriptives*							
**Time from fracture to surgery** (in days)		0.16 (−0.40, 0.71)	0.60 (0.05, 1.16)	0.17 (−0.33, 0.67)	0.62 (0.13, 1.12)	0.01	0.02
**Surgery instance**	1st surgery	0.67 (0.11, 1.23)	0.67 (0.12, 1.22)	3.16 (2.54, 3.79)	3.16 (2.57, 3.76)	2.49	2.49
	1st revision (2nd surgery)						
	2nd revision (3rd surgery)						
*Functional outcomes at discharge of inpatient rehabilitation* *(only participants who completed the inpatient rehabilitation program)*							
**FIM—Total Score**		0.46 (−0.10, 1.02)	0.42 (−0.14, 0.97)	0.48 (−0.00, 0.96)	0.44 (−0.03, 0.91)	0.02	0.02
**FIM—Motor Sub-Score**		0.43 (−0.13, 0.99)	0.37 (−0.19, 0.92)	0.46 (−0.02, 0.94)	0.40 (−0.07, 0.88)	0.03	0.03
**FIM—Cognition Sub-Score**		0.42 (−0.14, 0.98)	0.45 (−0.11, 1.00)	0.39 (−0.09, 0.86)	0.42 (−0.06, 0.89)	0.03	0.03
**TUG test time** (in seconds)		0.44 (−0.14, 1.02)	0.28 (−0.29, 0.86)	0.39 (−0.14, 0.92)	0.22 (−0.30, 0.75)	0.05	0.06
**Supportive device used during TUG test**	No gait device used	0.41 (−0.17, 0.99)	0.58 (0.00, 1.16)	0.38 (−0.15, 0.91)	0.58 (0.05, 1.10)	0.03	0.0
	Used 1–2 walking sticks						
	Used 1–2 crutches						
	Used rollator						
	Used walking frame						

^1^ Pairwise Standardized Mean Difference (SMD) with 95% Confidence Interval (CI). ^2^ FN-F: N = 118. ^3^ Periprosthetic fractures: N = 18. ^4^ Tr-F: N = 152. ^5^ Ac/Pp-F: N = 24.

**Table A10:** Individual paths are sorted into the four main healthcare pathway types.

*P1: From acute care directly to inpatient rehabilitation and discharge to outpatient setting*		*n*	*%*
1.	Independent Living → acute care hospital → inpatient rehabilitation → independent living	108	36.7
2.	Independent living → acute care hospital → inpatient rehabilitation → independent living with support or care	64	21.8
3.	Independent living with support or care → acute care hospital → inpatient rehabilitation → independent living with support or care	21	7.1
4.	Independent living → acute care hospital → inpatient rehabilitation → long-term care home	8	2.7
5.	Long-term care home → acute care hospital → inpatient rehabilitation → long-term care home	4	1.4
6.	Independent living with support or care → acute care hospital → inpatient rehabilitation → long-term care home	2	0.7
7.	Independent living → other → acute care hospital → other → inpatient rehabilitation → independent living with support or care	1	0.3
*P2: From acute care to temporarily care home followed by inpatient rehabilitation and discharge to outpatient setting*			
8.	Independent living → acute care hospital → temporary care home → inpatient rehabilitation → independent living with support or care	9	3.1
9.	Independent living with support or care → acute care hospital → temporary care home → inpatient rehabilitation → independent living with support or care	7	2.4
10.	Independent living → acute care hospital → temporary care home → inpatient rehabilitation → independent living	6	2.0
11.	Independent living → acute care hospital → independent living with support or care → inpatient rehabilitation → independent living with support or care	2	0.7
12.	Independent living → acute care hospital → independent living with support or care → inpatient rehabilitation → independent living	1	0.3
*P3: From acute care to inpatient rehabilitation and then to a temporarily care home*			
13.	Independent living → acute care hospital → inpatient rehabilitation → temporary care home → unknown	11	3.7
14.	Independent living with support or care → acute care hospital → inpatient rehabilitation → temporary care home → unknown	6	2.0
*P4: All individual paths with hospital re-admission irrespective of a temporary stay in a care home*			
15.	Independent living → acute care hospital → inpatient rehabilitation → acute care hospital → inpatient rehabilitation → independent living with support or care	8	2.7
16.	Independent living → acute care hospital → inpatient rehabilitation → acute care hospital → unknown	5	1.7
17.	Independent living with support or care → acute care hospital → inpatient rehabilitation → acute care hospital → unknown	3	1.0
18.	Independent living → acute care hospital → inpatient rehabilitation → acute care hospital → independent living	3	1.0
19.	Independent living → acute care hospital → inpatient rehabilitation → acute care hospital → inpatient rehabilitation → independent living	2	0.7
20.	Independent living → acute care hospital → independent living → acute care hospital → inpatient rehabilitation → independent living with support or care	2	0.7
21.	Independent living → acute care hospital → independent living → acute care hospital → inpatient rehabilitation → independent living	2	0.7
22.	Independent living with support or care → acute care hospital → unknown → acute care hospital → inpatient rehabilitation → independent living with support or care	1	0.3
23.	Independent living with support or care → acute care hospital → inpatient rehabilitation → acute care hospital → independent living with support or care	1	0.3
24.	Independent living with support or care → acute care hospital → inpatient rehabilitation → acute care hospital → inpatient rehabilitation → independent living with support or care	1	0.3
25.	Long-term care home → acute care hospital → unknown → acute care hospital → inpatient rehabilitation → acute care hospital → unknown	1	0.3
26.	Independent living with support or care → acute care hospital → inpatient rehabilitation → acute care hospital → inpatient rehabilitation → acute care hospital → unknown	1	0.3
27.	Independent living → acute care hospital → independent living → unknown → acute care hospital → inpatient rehabilitation → independent living	1	0.3
28.	Independent living → acute care hospital → inpatient rehabilitation → independent living with support or care → acute care hospital → inpatient rehabilitation → acute care hospital → inpatient rehabilitation → acute care hospital → unknown	1	0.3
29.	Independent living → acute care hospital → inpatient rehabilitation → acute care hospital → inpatient rehabilitation → temporary care home → unknown	1	0.3
30.	Independent living with support or care → acute care hospital → inpatient rehabilitation → acute care hospital → independent living with support or care → acute care hospital → inpatient rehabilitation → independent living with support or care	1	0.3
31.	Independent living → acute care hospital → inpatient rehabilitation → acute care hospital → inpatient rehabilitation → deceased	1	0.3
32.	Independent living → acute care hospital → inpatient rehabilitation → acute care hospital → long-term care home	1	0.3
33.	Independent living → acute care hospital → inpatient rehabilitation → acute care hospital → inpatient rehabilitation → acute care hospital → long-term care home	1	0.3
34.	Independent living → acute care hospital → temporary care home → acute care hospital → temporary care home → inpatient rehabilitation → independent living	1	0.3
35.	Independent living with support or care → unknown → temporary care home → acute care hospital → inpatient rehabilitation → acute care hospital → unknown	1	0.3
36.	Independent living → acute care hospital → inpatient rehabilitation → acute care hospital → inpatient rehabilitation → acute care hospital → inpatient rehabilitation → independent living with support or care	1	0.3
37.	Independent living → acute care hospital → inpatient rehabilitation → acute care hospital → inpatient rehabilitation → other → unknown	1	0.3
38.	Independent living → acute care hospital → other → unknown → acute care hospital → temporary care home → inpatient rehabilitation → independent living with support or care	1	0.3
39.	Independent living → acute care hospital → inpatient rehabilitation → independent living → acute care hospital → inpatient rehabilitation → independent living with support or care	1	0.3
40.	Independent living → acute care hospital → temporary care home → inpatient rehabilitation → acute care hospital → deceased	1	0.3

## Appendix B

Additional information and a detailed description of the variables in the appendix tables (Table A1, Table A2, Table A3, Table A4, Table A5, Table A6 and Table A7).

All variables were collected retrospectively from the medical reports and other entries from the medical information system from healthcare professionals, including mainly physicians, surgeons, physiotherapists, and nurses. Below, additional information is provided for variables in the appendix, which required further explanation and definitions.

### Appendix B.1. Appendix A Table A1 and Table A2

Baseline characteristics and variables referring to the pre-fracture situation include sociodemographic variables. Pre-fracture refers to the time before the first hospitalization.

Pre-fracture daily living and lifestyle variables included patient-reported sleep information, smoking status, alcohol consumption, and preparation of medication in the permanent living situation. These variables were assessed via patient self-report at admission to inpatient rehabilitation from healthcare professionals’ reports and reflect the period prior to hospitalization.

### Appendix B.2. Patient-Reported Sleep Information (Appendix A Table A1)

Sleep comprised two patient-reported dimensions. Sleep quality was categorized as follows: no sleep problems; sometimes or mild sleep problems; or permanent or severe sleep problems. Sometimes, mild sleep problems were considered if participants reported waking up occasionally at night, or having problems falling asleep. Permanent or severe sleep problems were chosen if participants reported having disturbed sleep every night due to not falling asleep, waking up too early/after only a few hours of sleep, or using the toilet several times at night. The two categories were chosen if participants did not compensate for sleep quality with medications.

Sleep medication use was categorized as follows: taking sleep medications (unspecified); taking Z-drugs (sedative–hypnotics); taking other medications for sleep (e.g., analgesics); using CPAP, BiPAP, or an oxygen mask; or as unknown. Taking sleep medications included, for example, Circadin, Melatonin Receptor Agonists, or Antidepressants. Taking Z-drugs included medications like Zolpidem, Benzodiazepines, or Quetiapine.

### Appendix B.3. Patient-Reported Alcohol Consumption (Appendix A Table A1)

Alcohol consumption was categorized as follows: no alcohol consumption; mild-to-moderate consumption; abusive consumption; or unknown. Mild-to-moderate drinking was chosen when participants reported to drink sometimes, occasionally, in social situations, or one/a glass to a meal. Abusive drinking was considered when participants reported to drink daily more than one unit (one unit would be one appropriate glass). Participants reported for example to drink more than one bottle of beer or wine per day, several glasses of an alcoholic drink, or admitted to have an abusive drinking behavior.

### Appendix B.4. Walking Ability (Appendix A Table A2)

The categorization was adopted from the Geriatric Trauma Registry of the German Society for Orthopedics and Trauma Surgery (in German «Alters-Trauma-Register (ATR) der Deutsche Gesellschaft für Orthopädie und Unfallchirurgie (DGU)») [58]. Walking ability was derived from the physiotherapists’ reports.

### Appendix B.5. Usage of Assistive Gait Devices (Appendix A Table A2)

Assistive gait devices were collected from the therapists’ reports at the time of admission to the rehabilitation center. The variable allowed multiple selections per participant.

### Appendix B.6. Experience of Gait and Balance Disturbances (Table 1)

Gait and balance disturbances were collected by reviewing the medical diagnosis list from the acute care hospital and rehabilitation.

### Appendix B.7. Experienced Previous Low-Trauma Fracture(s) at the Hip, Pelvis, Proximal Femur, or Femoral Shaft and Experienced Previous Low-Trauma Fracture(s) at Other Locations (Table 1 and Appendix A Table A3)

These two variables were collected by reviewing medical diagnosis lists and reports. If nothing was documented, or if it was unclear whether the fracture was low-trauma, the value was recorded as “unknown”.

### Appendix B.8. Mild Cognitive Impairment, Early Stage of/Highly Suspected Dementia, and/or Other Cognitive Difficulties (Appendix A Table A3)

This variable was collected by reviewing medical diagnosis lists and reports.

The other co-morbidity variables were collected from the medical diagnosis list by categorizing the following ICD codes (Appendix B.9, Appendix B.10, Appendix B.11, Appendix B.12, Appendix B.13, Appendix B.14, Appendix B.15, Appendix B.16, Appendix B.17, Appendix B.18, Appendix B.19, Appendix B.20, Appendix B.21, Appendix B.22, Appendix B.23, Appendix B.24, Appendix B.25, Appendix B.26, Appendix B.27, Appendix B.28, Appendix B.29, Appendix B.30, Appendix B.31, Appendix B.32, Appendix B.33 and Appendix B.34; Appendix A Table A3):

### Appendix B.9. Osteoporosis

M80, M81, and M82.

Note: Osteoporosis diagnoses were taken from the list of diagnoses and were considered to be confirmed diagnoses according to previous definitions (where a low-trauma fracture was not sufficient for fully filling the criteria for osteoporosis).

### Appendix B.10. Osteoarthritis and Other Arthropathies

M00-M03, M05-M14, M15, M16, M17, M18, M19, and M20-M25.

### Appendix B.11. Other Diseases of the Musculoskeletal System, and Soft and Connective Tissue

M30-M36, M60-M63, M65-M68, M70-M79, and M95-M99.

### Appendix B.12. Spondylopathies and Other Spine Related Diseases

M40-M43, M45-M49, and M50-M54.

### Appendix B.13. Dementia

F00-F03, G30, G31.0, and G31.1.

### Appendix B.14. Substance-Use Disorders

F10-F19, F55, K70, G31.2, and G62.1.

### Appendix B.15. Other Psychiatric Disease and Mental Health Diseases

F04, F06, F07, F09, F20-F25, F28-F34, F38-F45, F48, F50-F54, F59-F66, F68-F74, F78-F84, F88-F95, F98, and F99.

### Appendix B.16. Polyneuropathies

G60-G63.

### Appendix B.17. Parkinson, Multiple Sclerosis, Restless Leg Syndrome, and Other Extra Pyramidal Diseases

G20-G26 and G35.

### Appendix B.18. Diabetes Mellitus

E10-E14.

### Appendix B.19. Thyroid and Parathyroid Diseases

E00-E07 and E20-E21.

### Appendix B.20. Vitamin D and Folic Acid Deficiencies

E55, D52, and E53.8 (if folic acid was labeled for the code E53.8).

### Appendix B.21. Malnutrition and Other Anemia

E40-E46, E50-E64, D50-D53 (excluding D52 and E53.8 for folic acid), and K90.

### Appendix B.22. Adiposities and Overnutrition

E65-E68.

### Appendix B.23. Lipidemia Including Hyperlipidemia

E78.

### Appendix B.24. Other Metabolic and Nutritional Disruption

E15-E16 and E70-E90 (excluding E78).

### Appendix B.25. Coronary Heart Diseases

I25.

### Appendix B.26. Congestive Heart Failure

I50.

### Appendix B.27. Arrhythmia and Conduction Disorders

I44-I49.

### Appendix B.28. Other Ischemic Heart and Other Forms of Heart Disease

I20-I24, I30-I43, and I51-I52.

### Appendix B.29. Hypertensive Diseases

I10-I13 and I15.

### Appendix B.30. Arterial Diseases and Other Cardiovascular

I70-I74, I77-I83, I85-I89, I95, and I97-I99.

### Appendix B.31. Chronic Obstructive Pulmonary Disease

J44.

### Appendix B.32. Renal Insufficiency

N17-N19.

### Appendix B.33. Malignant Tumor Without Metastasis

C00-C75, C76, C81-C96, D00-D09, D37-D48, and C97.

### Appendix B.34. Malignant Tumor with Metastasis

C77-C80.

### Appendix B.35. Length of First Stay in Acute Care Hospital (Appendix A Table A4)

The length of the first acute care hospital stay was defined as the initial hospitalization following the fracture and was calculated in days from the admission and discharge dates, excluding any re-admissions.

### Appendix B.36. American Society of Anesthesiologists (ASA) Grading (Appendix A Table A4)

The ASA score was retrospectively assessed from the medical reports. In the table, the levels Grade I and Grade V were left out because no participant was assigned to these levels [60].

### Appendix B.37. Clavien–Dindo Post-Surgical Complications Classification (Appendix A Table A5)

The variable was retrospectively assessed from the medical reports. In the table, the level Grade V (death) was left away because no participant was assigned to this level [61].

### Appendix B.38. Number of Medications (Appendix A Table A6)

The number of medications was assessed from the medication report at the time of admission to the rehabilitation center.

### Appendix B.39. Pain Numerical Rating Scale (Appendix A Table A6)

At the time of admission to the rehabilitation center, participants reported perceived pain on a scale from 0 to 10 (with 0 no pain and 10 most imaginable pain).

### Appendix B.40. Nutritional Risk Screening (Appendix A Table A6)

The Nutritional Risk Screening score was assessed at admission the time of admission to the rehabilitation by nutritional therapists. The score ranges from 0 to 7. The Nutritional Risk Screening instrument is based on Kondrup et al. [62].

### Appendix B.41. Geriatric Depression Scale, Mini Mental State Examen, and Clock Test (Appendix A Table A6)

These assessments were only assessed for individuals participating in a specialized musculoskeletal–geriatric rehabilitation program or if clinically indicated [63,64,65,66]. For the participants undergoing MSK–geriatric rehabilitation, the score was assessed within the first week of stay.

For the Geriatric Depression Scale, two versions were used: the short version, with 15 items, and the brief version, with five items. The brief version was used from September 2022, before the short version was used [63,66].

### Appendix B.42. Length of Stay in Rehabilitation (Appendix A Table A6)

Total length of stay in inpatient rehabilitation was calculated in days from admission and discharge dates, and includes re-admission stays.
